# Exosomes produced by melanoma cells significantly influence the biological properties of normal and cancer-associated fibroblasts

**DOI:** 10.1007/s00418-021-02052-2

**Published:** 2021-11-27

**Authors:** Karolína Strnadová, Lucie Pfeiferová, Petr Přikryl, Barbora Dvořánková, Erik Vlčák, Jana Frýdlová, Martin Vokurka, Jiří Novotný, Jana Šáchová, Miluše Hradilová, Jan Brábek, Jana Šmigová, Daniel Rösel, Karel Smetana, Michal Kolář, Lukáš Lacina

**Affiliations:** 1 Institute of Anatomy, 1st Faculty of Medicine, Charles University, 128 00 Prague 2, Czech Republic; 2 BIOCEV, 1st Faculty of Medicine, Charles University, 25250 Vestec, Czech Republic; 3 Laboratory of Genomics and Bioinformatics, Institute of Molecular Genetics of the Czech Academy of Sciences, 142 20 Prague 4, Czech Republic; 4 Institute of Pathological Physiology, 1st Faculty of Medicine, Charles University, 128 00 Praha, Czech Republic; 5 Electron Microscopy Core Facility, Institute of Molecular Genetics of the Czech Academy of Sciences, 142 20 Prague 4, Czech Republic; 6 BIOCEV, Faculty of Sciences, Charles University, 25250 Vestec, Czech Republic; 7 Department of Dermatovenereology, 1st Faculty of Medicine, Charles University and General University Hospital, 120 00 Prague 2, Czech Republic; 8 Department of Informatics and Chemistry, Faculty of Chemical Technology, University of Chemistry and Technology, Prague, Czech Republic

**Keywords:** Melanoma, Cancer-associated fibroblasts, Exosomes, IL-6, IL-8, Proinflammatory cytokine

## Abstract

The incidence of cutaneous malignant melanoma is increasing worldwide. While the treatment of initial stages of the disease is simple, the advanced disease frequently remains fatal despite novel therapeutic options . This requires identification of novel therapeutic targets in melanoma. Similarly to other types of tumours, the cancer microenvironment plays a prominent role and determines the biological properties of melanoma. Importantly, melanoma cell-produced exosomes represent an important tool of intercellular communication within this cancer ecosystem. We have focused on potential differences in the activity of exosomes produced by melanoma cells towards melanoma-associated fibroblasts and normal dermal fibroblasts. Cancer-associated fibroblasts were activated by the melanoma cell-produced exosomes significantly more than their normal counterparts, as assessed by increased transcription of genes for inflammation-supporting cytokines and chemokines, namely IL-6 or IL-8. We have observed that the response is dependent on the duration of the stimulus via exosomes and also on the quantity of exosomes. Our study demonstrates that melanoma-produced exosomes significantly stimulate the tumour-promoting proinflammatory activity of cancer-associated fibroblasts. This may represent a potential new target of oncologic therapy .

**Supplementary Information:**

The online version contains supplementary material available at 10.1007/s00418-021-02052-2.

## Introduction

Cancer incidence has reached a pandemic-like extent in numerous developed countries worldwide. The explanation for this phenomenon is not straightforward: numerous factors ranging from environmental pollution and food contamination, through excessive caloric intake to civilisation-associated stressful lifestyle seem to contribute to this trend. Another critical factor is ageing of the population (Smetana et al. 2016). Cutaneous malignant melanoma (CMM) follows the trend of other cancers. Moreover, climate and lifestyle changes represent another important contribution to the increasing incidence of CMM, as tumour initiation and progression is strongly facilitated by ultraviolet (UV) irradiation (Jobe et al. 2018).

Despite remarkable progress in CMM-targeted therapy and immunotherapy, the metastatic disease represents a therapeutic challenge and leaves patients with an uncertain prognosis (Smetana et al. 2020). From this perspective, identification of new therapeutic targets is critical to manage CMM successfully (Brustugun et al. 2014). Similarly to other types of tumours, CMM is not only composed of cancer cells, but also contains other cell types. The tumour forms a malignant organ that evolves in the patient’s body (Alberts et al. 2002; Egeblad et al. 2010). Among non-cancerous cells forming the cancer ecosystem within the tumour niche, one has to emphasise the prominent role of different subtypes of immune cells and also cancer-associated fibroblasts (CAFs) (Lacina et al. 2015; Falcone et al. 2020). These non-malignant cells can substantially influence the biological properties of cancer cells, resulting in their locally aggressive growth and increased migratory potential leading to metastatic spread (Kodet et al. 2020). The cells shaping the landscape of the tumour microenvironment must be precisely coordinated, similarly to cell populations forming a normal organ. Cancer cells and the surrounding stromal elements communicate by their mutual intercellular contacts, in an autocrine and paracrine signalling manner. It necessitates production of bioactive agents that include extracellular matrix molecules, growth factors, cytokines and chemokines, frequently with properties supporting chronic inflammation. A distinct position in the regulation of the cancer ecosystem is held by IL-6 and IL-8 (Jobe et al. 2016; Brábek et al. 2020).

The role of extracellular vesicles, including exosomes, as versatile communication tools mediating intercellular interactions is well accepted (Zebrowska et al. 2020), and they represent a significant component of the tumour microenvironment in cancer biology. The activity of exosomes depends on their cargo. It is formed by bioactive proteins (e.g., PD-L1, IL-6, IL-10, TNF-α, TGF-β1) and a broad range of microRNAs or mRNAs that can control the functional properties of targeted cells (Zebrowska et al. 2020). As expected, these substances were also detected in biological fluids of melanoma patients (Pfeffer et al. 2015; Kučera et al. 2019). All cell populations forming the tumour ecosystem secrete extracellular vesicles, and their activity is broad. Exosomes, via their cargo, can modulate functions of cancer stem cells, cancer-associated fibroblasts, immune cells, and endothelium.

Interestingly, exosomes deliver cargoes that can both be of cancer-supporting and cancer-inhibiting nature (Cavallari et al. 2020). The melanoma-produced exosomes promote proinflammatory signalling important for tumour local progression and metastasis (Gener Lahav et al. 2019; Kodet et al. 2020). Moreover, exosomes can suppress the antitumoural activity of infiltrating CD8 + T lymphocytes (Shu et al. 2020). Further, melanoma-derived exosomes have a distinct role in the transformation of fibroblasts to cancer-associated fibroblasts (la Shu et al. 2018; Hu and Hu 2019). All these findings have highlighted the prominent role of exosomes in the tumour microenvironment. Therefore, exosomes are in the focus of interest of clinical oncology and represent a potential therapeutic target in anticancer research.

We need to understand the contribution of exosomes in the progression of malignant melanoma within the tumour microenvironment and in the course of metastatic spread in the organism. In this study, we aimed to demonstrate in vitro the different effect of melanoma-produced exosomes on the functional properties of normal dermal fibroblasts and cancer-associated fibroblasts isolated from CMM (mCAFs). This knowledge could be critically important for deeper insight into the mechanisms supporting local progression and metastatic spread to remote body sites. It also opens further discussion on whether exosomes in the tissue microenvironment and circulating in body fluids could serve as biomarkers for melanoma diagnosis and disease prognosis.

## Material and methods

### Cell culture for exosome isolation

Authenticated melanoma cell line G361 (CRL-1424, ATCC, Manassas, VA, USA) was chosen as an efficient exosome producer. The cells were routinely maintained in McCoy’s 5A medium (BioConcept, Allschwil, Switzerland) with antibiotics (100 × concentrated penicillin and streptomycin) and with 10% of foetal bovine serum (FBS, both Biosera, Nuaille, France). Six million G361 cells were inoculated to each of eight 150-cm^2^ flasks (Corning, Glendale, AZ, USA) in McCoy’s 5A medium with 10% FBS for 24 h. For exosome harvesting, we replaced the medium using 35 ml of Dulbecco’s modified Eagles medium (DMEM, Biosera, Nuaille, France) with 5% exosome-depleted foetal bovine serum (EDS, A27208-01, Thermo Fisher Scientific, Waltham, MA, USA). We kept the flasks for the next 72 h in a humidified incubator (37 °C, 5% CO_2_). From eight 150-cm^2^ flasks, we collected a total of 280 ml of conditioned growth medium for exosome isolation.

Other tested melanoma cell lines, A2058 (CRL-11147, ATCC, Manassas, VA, USA) and BLM (a generous gift provided by Prof. JHJM van Krieken, Radboud University, Nijmegen, Netherlands) (Quax et al. 1991) were maintained in DMEM supplemented by 10% FBS and used as above. All experiments were performed in three independent replicates.

To test the non-specific effect of extracellular vesicles (unrelated to melanoma) as an additional control for some of our experiments, we also isolated exosomes from FBS used for cell culture.

### Exosome isolation and characterisation

#### Exosome isolation by density gradient ultracentrifugation

Exosomes were isolated by cushioned-density gradient ultracentrifugation as previously described by Li et al. with partial modifications (Li et al. 2018). This protocol maximises exosome recovery and allows for better preservation of their physical integrity and biological activity. Briefly, the conditioned medium was centrifuged at 300×*g* for 10 min at 4 °C to pellet cells and debris. The supernatant was then centrifuged at 2000×*g* for 15 min at 4 °C, transferred to new tubes, centrifuged for 30 min at 10,000×*g* at 4 °C and filtered through a 0.22 μm Steriflip-GP sterile filter (Merck Millipore, Burlington, MA, USA). Then, six aliquots of 28 ml of this cleared conditioned media (CM) were underlain with 2 ml of 60% OptiPrep density gradient medium (Alere Technologies AS, Oslo, Norway) and concentrated in ultracentrifuge tubes using cushioned ultracentrifugation at 140,000×*g* for 3 h with an SW 28 Ti swinging-bucket rotor in an Optima XPN 80 ultracentrifuge (Beckman-Coulter, Brea, CA, USA). Two ml of Optiprep and 1 ml of supernatant above the cushion were aspirated from the bottom by a blunt-point needle to obtain 3 ml of cushioned concentrate per tube. Concentrates were placed to the bottom of the tubes below a discontinuous gradient composed of the top 3 ml of 5%, then 3 ml of 10%, and 3 ml of 20% Optiprep solution. The ultracentrifuge tubes with the gradient were then spun at 140,000×*g* for 18 h at 4 °C using an SW 28.1 Ti swinging-bucket rotor. After centrifugation, the tube content was divided into 12 1 ml fractions and collected starting from the top of the tube. Individual fractions nos. 6, 7 and 8, containing purified exosomes, from the six tubes were pooled together, diluted with filtered phosphate-buffered saline (PBS) and re-centrifuged again at 140,000×*g* for 2 h at 4 °C. Finally, these washed and concentrated fractions were resuspended in 50 μl of PBS, combined, filled to a volume of 200 μl in a protein LoBind tube (Eppendorf, Hamburg, Germany) and stored at 4 °C or −80 °C. Buoyant densities of the gradient fractions were estimated by measuring the absorbance at 340 nm using a multi-well plate reader. Contamination of fractions with non-exosomal proteins was monitored using the Qubit Protein Assay Kit and a Qubit 2.0 fluorometer (Thermo Fisher Scientific, Waltham, MA, USA) (Li et al. 2018). Exosomes present in fractions 6–8 were confirmed by western blot, transmission electron microscopy and nanoparticle tracking analysis.

#### Detection of exosome protein concentration

For the purposes of experiment normalisation, the quantity of exosomes was determined as the total protein concentration using a Pierce BCA Protein Assay Kit (23,225 Thermo Fisher Scientific, Waltham, MA, USA). Briefly, exosomes were disrupted using 25% Triton X-100 in PBS (25 µl exosome suspension and 1 µl Triton) by agitation on a shaker (300 RPM) at room temperature for 15 min. Then, the sample was processed following the manufacturer’s instructions. Absorbance was measured at 562 nm using a SpectraMax iD3 (Molecular Devices, San Jose, CA, USA), and the concentration of total protein was extrapolated from the calibration curve. For cell culture applications, we used exosomes in a quantity corresponding to 10 µg/ml of total protein diluted in culture medium (unless otherwise specified).

#### Exosome characterisation by SDS-PAGE and western blot analysis

Exosomal fractions were supplemented with a protease inhibitor cocktail and lysed for at least 15 min in RIPA buffer. We followed by adding 4 × Laemmli sample buffer (Bio-Rad, Hercules, CA, USA) and boiling at 95 °C for 5 min. Proteins (2 μg per well) were loaded to 12% SDS polyacrylamide gel and electrophoresed at 125 V for 70 min. Separated proteins were transferred from gels onto 0.45 μm PVDF membranes at 20 V for 60 min. The membranes were blocked in 5% skim milk in TBS containing 0.1% Tween 20 for 1 h and probed overnight at 4 °C in blocking solution containing primary antibodies against classical exosome markers CD9, CD63 and CD81 (all Thermo Fisher Scientific, Waltham, MA, USA—see Supplementary Table 1). Then, the membranes were washed five times for 5 min in TBS-Tween buffer and incubated at room temperature for 1 h with HRP-conjugated secondary antibody. Probed membranes were then washed five times in TBS-Tween buffer. Chemiluminescent detection using LumiGLO Reagent (Cell Signaling Technology) was performed in the ChemiDoc MP Imaging System (Bio-Rad, Hercules, CA, USA).

#### Exosome characterisation by nanoparticle tracking analysis (NTA)

The size and concentration of extracellular vesicles were analysed by NTA using the NanoSight NS300 instrument (Malvern Panalytical, Malvern, Great Britain). To achieve a concentration range of 10^8^ − 10^9^ particles per ml, isolated exosome fractions were diluted in PBS filtered through a 0.1 μm Stericup filter. Each sample was analysed with a optimised auto-setup camera level and a detection threshold value of 5. Five replicate video recordings of 180-s duration per fraction were collected. Statistical analysis and graph plotting of resulted data was performed with NanoSight NTA software version 3.2.

### Electron microscopy of exosomes

#### Ultrathin sections of collagen-embedded exosomes and their electron microscopic analysis

Melanoma-derived exosomes were mixed with neutralised rat tail collagen-1 (2 mg/ml; Advanced BioMatrix, San Diego, CA, USA) to reach the final exosome protein concentration of 10 µg/ml and cast into a cap of a 0.5 ml Eppendorf tube. The polymerised collagen block with embedded exosomes was removed and fixed using 2.5% glutaraldehyde in 0.1 M Sörensen’s sodium–potassium phosphate buffer (SB) with adjusted pH 7.2–7.4 (all Thermo Fisher Scientific, Waltham, MA, USA). After several gentle washes in SB, samples were postfixed with 1% osmium tetroxide in SB for 2 h at room temperature. Samples were then dehydrated in a series of acetone (Lach-Ner, Neratovice, Czech Republic) with increasing concentration and embedded in Epon-Durcupan resin (Sigma-Aldrich, St. Louis, MO, USA). After polymerisation for 72 h at 60 °C, resin blocks with embedded samples were cut into 80 nm ultrathin sections using a Ultramicrotome Leica EM UC6 (Leica Microsystems, Wetzlar, Germany) with a diamond knife (Diatome, Biel, Switzerland) and mounted on 200 mesh size copper grids. Sections were examined in a JEOL JEM-1400Flash transmission electron microscope operated at 80 kV equipped with a Matataki Flash sCMOS camera (JEOL, Akishima, Tokyo, Japan).

#### Negative staining of isolated exosomes

Undiluted suspensions were applied to glow-discharged 400 mesh size copper grids with thin formvar/carbon film and allowed to absorb for 20 min. Excess was blotted away with filter paper, and the absorbed sample was fixed in a drop of 1% formaldehyde (all Thermo Fisher Scientific, Waltham, MA, USA) for 20 min. Grids were then quickly washed in double-distilled water and negatively stained with 2% methylcellulose (Sigma-Aldrich, St. Louis, MO, USA) and 0.4% uranyl acetate (Honeywell, Charlotte, NC, USA) for 10 min. Excess solution was blotted away, and grids were air-dried. Samples were examined in a JEOL JEM-1400Flash transmission electron microscope operated at 80 kV equipped with a Matataki Flash sCMOS camera (JEOL, Akishima, Tokyo, Japan).

#### Establishment of fibroblast cell lines

Fibroblasts were isolated as published elsewhere (Dvořánková et al. 2019). For this purpose, we used two different fibroblast types, mCAFs and human dermal fibroblasts (HDFs); two biological replicates of each type were used in all experiments. Briefly, these fibroblasts were isolated earlier from the residual tissue from the operation, and both samples were of the dermal origin from the trunk region. Tissues were collected after local ethics committee approval following the ethical standards of the Institutional and National Research Committee and according to the 1964 Helsinki Declaration and its later amendments or comparable ethical standards. The patient’s informed consent was obtained.

### Transcriptome analysis

#### Transcriptome analysis after 24 h of cultivation

Cell suspensions were inoculated in DMEM + 10% foetal bovine serum and cultured for 24 h. The next day, the medium was replaced by DMEM + 10% EDS without/with G361-derived exosomes. The cells were cultured for an additional 24 h. Cells were washed in PBS and digested in 0.25% trypsin + EDTA solution (1:1) (all Sigma-Aldrich, St. Louis, MO, USA) at room temperature. The viability of cells was assessed by trypan blue and counted in an automated TC20 cell counter (Bio-Rad, Hercules, CA, USA). Both samples had cell viability above 99%.

Single-cell RNA-seq libraries were prepared using a Chromium controller instrument and Chromium Next Gem single-cell 3’ reagent kit (version 3.1) according to the manufacturer’s protocol (both 10 × Genomics, Pleasanton, CA, USA) targeting at 4000 cells per sample. The quality and quantity of the resulting cDNA and libraries were determined using an Agilent 2100 Bioanalyzer (Agilent Technologies, Santa Clara, CA, USA). The libraries were sequenced in two runs of a NextSeq 500 sequencer using a NextSeq 500/550 high output kit v2.5 (75 cycles) (both Illumina, San Diego, CA, USA) according to the manufacturer’s protocol.

Raw sequencing data were processed by CellRanger software v. 4.0.0 (10 × Genomics, Pleasanton, CA, USA). The resulting raw feature barcode matrices were analysed in the R/Bioconductor statistical environment (R Core Team 2020). Empty droplets containing only ambient RNA were removed using DropletUtils (Lun et al. 2018). Subsequently, dead or damaged cells were filtered out, and features expressed in less than 5% of cells were removed from the barcode matrix, resulting in 4207, 3955, 2390 and 3098 cells in the HDFs control, HDF EXO G361, mCAFs control and mCAFs EXO G361 sample, respectively, and, in the same sample order, 11,832, 11,796, 11,860 and 11,918 features. The data were normalised, log2-transformed, and features within each sample were averaged to produce pseudo-bulk expression values. Heatmaps were produced by the ComplexHeatmap package (Gu et al. 2016).

All transcriptomic data were deposited in the ArrayExpress database (http://www.ebi.ac.uk/arrayexpress) under accessions E-MTAB-10278 and E-MTAB-10290.

### Transcriptome analysis after 72 h of cultivation

Both fibroblast types (mCAFs and HDFs) were inoculated in DMEM with 10% FBS at the density of 3500 cells/cm^2^ in 6-well plates. After 24 h, culture medium was changed for: (a) DMEM with 10% exosome-depleted serum (EDS, Gibco/ Thermo Fisher Scientific, Waltham, MA, USA) (control); (b) DMEM with 10% EDS + G361-derived exosomes; and (c) DMEM with 10% EDS + exosomes from FBS (also 10 µg/ml), and the cells were cultured for 72 h. Then, the cells were washed twice with PBS, and the cell lysates were harvested by 350 µl buffer RLT (Qiagen GmbH, Hilden, Germany) and 2-mercaptoethanol (Sigma-Aldrich, St. Louis, MO, USA; Merck KGaA, Darmstadt, Germany), immediately frozen and stored at −80 °C. Total RNA was isolated from 180 μl of the cell lysate according to the RNeasy Micro Kit (Qiagen GmbH, Hilden, Germany) manufacturer’s protocol, including treatment by DNase I. The quantity and quality of isolated RNA were measured by a NanoDrop ND-1000 (Thermo Fisher Scientific, Waltham, MA, USA) and Agilent 2100 Bioanalyzer (Agilent Technologies, Santa Clara, CA, USA). The RNA integrity number (RIN) ranged between 8.6 and 10. For the sequencing library construction, a KAPA mRNA HyperPrep kit with poly(A) mRNA selection (Roche, Basel, Switzerland) was used, starting with 1 μg of total RNA. Libraries were sequenced on a NextSeq 500 platform (Illumina, San Diego, CA, USA) using the 75-bp single-end configuration. The sequencing yielded an average of 30 million reads per sample.

Technical quality control and gene quantification were done using the nf-core/rnaseq v2.0 bioinformatics pipeline (Ewels et al. 2020), with HISAT2 mapping (Kim et al. 2015) and featureCounts read counting (Liao et al. 2014). GRCh38 (Ensembl assembly version 95) was chosen as the reference genome (Yates et al. 2020). Genes expressed in a single sample only were discarded. The DESeq2 (v1.30.1) (Love et al. 2014) Bioconductor (v3.12) R package was used to identify differentially expressed genes. Significant changes in gene expression were defined by two-fold change and false discovery rate (FDR) < 0.1. Shrunken log-fold change estimates were used (adaptive shrinkage estimator; Stephens 2017). The gene set enrichment analysis was performed in Gene Ontology terms (Ashburner et al. 2000) using the goseq package (Young et al. 2010). The mevalonate pathway was visualised using PathView software (Luo and Brouwer 2013).

### Proteomic analysis of cultured medium and ELISA

For proteomic analysis, mCAFs and HDFs (3 500 cells/cm^2^) were cultured in (a) DMEM with 10% EDS (control) or (b) DMEM with 10% EDS + G361-derived exosomes. After 72 h, we collected the conditioned culture media for proteomic analysis using a Proteome Profiler Human XL Cytokine Array (R&D Systems, Minneapolis, MN, USA). Briefly, we used the membrane-based antibody array for parallel determination of the relative levels of selected human cytokines and chemokines according to the manufacturer’s instructions. Freshly prepared conditioned media were centrifuged to remove the debris and incubated overnight with the Proteome Profiler™ Human XL Cytokine Array membrane. The membranes were washed to remove unbound analytes, followed by incubation with a cocktail of biotinylated detection antibodies. Streptavidin-HRP and chemiluminescent detection reagents provided by the manufacturer were then applied. Finally, the signal produced at each capture spot corresponding to the amount of protein bound was detected using a C-DiGit Blot Scanner and analysed using the Image Studio software (LI-COR Biosciences GmbH, Bad Homburg, Germany).

Quantification of IL-6 and CXCL-8 was performed according to protocol using the AssayMax™ Human IL-6 ELISA Kit and AssayMax™ Human Interleukin 8 (IL-8) ELISA Kit (both BioVendor, Brno, Czech Republic).

### Cell culture of mCAFs and HDFs influenced by G361-derived exosomes

#### Evaluation of the effect of exosomes on cell proliferation and MTT assay

Using the iCELLigence instrument (Agilent Technologies, Santa Clara, CA, USA), we studied the growth of fibroblasts influenced by exosomes. Briefly, we seeded 5000 fibroblasts in DMEM with 10% EDS to each well (0.7 cm^2^) in a plate compatible with iCELLigence (two biological replicates of each, three technical experiments per run). Then, we added the suspension of exosomes. To determine efficient exosome concentration, we used a scale of concentrations (data obtained with 3 µg/ml and 10 µg/ml are presented). We used Dulbecco’s PBS (BioConcept, Allschwil, Switzerland) for control experiments. The final volume of the culture medium was equalised to 450 µl in each well. We monitored the cell proliferation for the next 96 h. Finally, we evaluated the viability of cells by MTT in the wells (Mosmann 1983).

#### Evaluation of the effect of exosomes on HDF and mCAF adhesion and migration (velocity) in 2D and 3D

Cell adhesion area measurement was performed by monitoring electrical impedance (cell index value) in real time using the xCELLigence RTCA System (Agilent Technologies, Santa Clara, CA, USA). Cells were incubated in DMEM with 10% EDS + G361-derived exosomes on a rotator for 4 h at 37 °C and then seeded into a pre-treated xCELLigence plate (Hamidi et al. 2017). Cell adhesion was monitored every 2 min for the first 30 min and every 5 min for the next 90 min. Further, measurement was performed every 15 min for the next 11 h.

Cell migration was studied using time-lapse microscopy of cells seeded in glass-bottom Ibidi 8-well plates (Ibidi GmbH, Gräfelfing, Germany) coated with fibronectin. Cells were incubated with G361-derived exosomes for 8 h and afterwards recorded at 15-min intervals for 13 h. The images were captured as tile scans using a Leica Thunder system (Leica Microsystems, Wetzlar, Germany) equipped with an LAS-X Navigator software module. Data analysis was done using ImageJ/Fiji in three consecutive sets of steps (data not shown). First, the collected series of images were stitched using the Stitching plug-in of ImageJ/Fiji. Second, the stitched imaged were processed for cell visualisation using the Edge Finder Tool, background suppression and binary masking. Third, cell tracking was performed at 16 time points with ~ 55 cells using the TrackMate plug-in of ImageJ/Fiji. All experiments were performed in parallel with a control (DMEM with 10% EDS) and exosome-treated (DMEM with 10% EDS and exosomes) mCAFs and HDFs. The step-by-step summary of this protocol is presented in Supplementary Fig. 5 with minimal and maximal velocity assessment.

The mobility of HDFs and mCAFs treated with exosomes was also evaluated using a 3D spheroid assay. 3D fibroblast spheroids were formed of ~ 700 cells an in agarose mould for 72 h. The spheroids were embedded into rat tail collagen (1 mg/ml) and covered with DMEM + 10% EDS with or without G361-derived exosomes. The images were captured at time 0, 24 and 48 h using a Leica Thunder system. The spheroid area was outlined using the Threshold or Edge Finder tool of ImageJ/Fiji. The invasion index was calculated as a normalised ratio of the spheroid area of interest to the starting spheroid area. The normalisation was done to the average spheroid area of control cells measured at time 0 h.

Statistical significance was evaluated using GraphPad Prism software (version 8.0.0 for Windows, GraphPad Software, San Diego, CA, USA, www.graphpad.com) by a two-tailed Student’s *t* test after ascertaining normality of the data (*p* value ≤ 0.05).

#### Evaluation of the effect of G361-derived exosomes on 3D melanoma spheroid invasion

Heterogeneous spheroids were prepared from melanoma cell line G361 with HDFs or mCAFs using the hanging drop method (Novotný et al. 2020). Briefly, mixed-cell spheroids were formed of 50,000 cells in a 1:1 ratio. Drops of mixed-cell suspension (each 25 µl) were placed on the inner side of a 100 mm Petri dish lid for 48 h, while the bottom of the dish was filled with 15 ml of PBS (Biosera, Nuaille, France). Afterwards, spheroids were gently transferred by a Pasteur pipette to non-adhesive Petri dishes with complete culture medium for an additional 24 h. To test the invasion, heterogeneous spheroids were transferred onto 1% low-melting-point agarose-coated dishes (Promega, Madison, WI, USA) and covered with neutralised collagen-1 from rat tails (2 mg/ml; Advanced BioMatrix, CA, USA) with or without G361-derived exosomes. For optimal stability during the extended experiment, the polymerised collagen was overlaid with 1% low-melting-point agarose. Finally, it was gently overlaid with DMEM + 10% EDS. The culture medium was changed every 48 h for 1 week. The endpoint images were captured after 144 h using an Olympus IX71 (Olympus, Shinjuku, Japan). The spheroid area was outlined using the Threshold and Edge Finder plug-in of ImageJ/Fiji. The invasion index was calculated as a normalised ratio of the spheroid of interest to the final outgrowth area to the initial spheroid area. Statistical significance was tested as above.

### Immunocytochemistry

Fibroblasts were seeded in the µ-Slide 8 Well system (Ibidi GmbH, Gräfelfing, Germany) at a density of 10,000/cm^2^ in DMEM + 10% EDS with or without G361-derived exosomes. Cells were fixed in 2% paraformaldehyde and permeabilised by TBS with 0.2% Tween. Endogenous peroxidase was blocked by incubation with 3% hydrogen peroxide in TBS at room temperature for 20 min. To block non-specific protein binding and primary antibody dilution, we used Universal IHC Blocking/Diluent (Leica, Wetzlar, Germany). After overnight incubation at 4 °C, the slides were washed, and the immunohistochemical reaction was developed using Histofine High Stain™ HRP (MULTI) and AEC substrate (both Nichirei Biosciences Inc, Tokyo, Japan). Slides were counterstained in Gill’s haematoxylin and mounted in Hydromount. Negative controls were performed using isotype control antibodies (Thermo Fisher Scientific, Waltham, MA, USA). The imaging was performed using the Leica DM2000 system (Leica Microsystems, Wetzlar, Germany) equipped with the LAS software. Primary antibodies were validated by producers and provided proof of validation on the technical specifications. All antibodies are listed in Supplementary Table 1.

## Results

### Characterisation of melanoma-derived exosomes

**Fig. 1:**
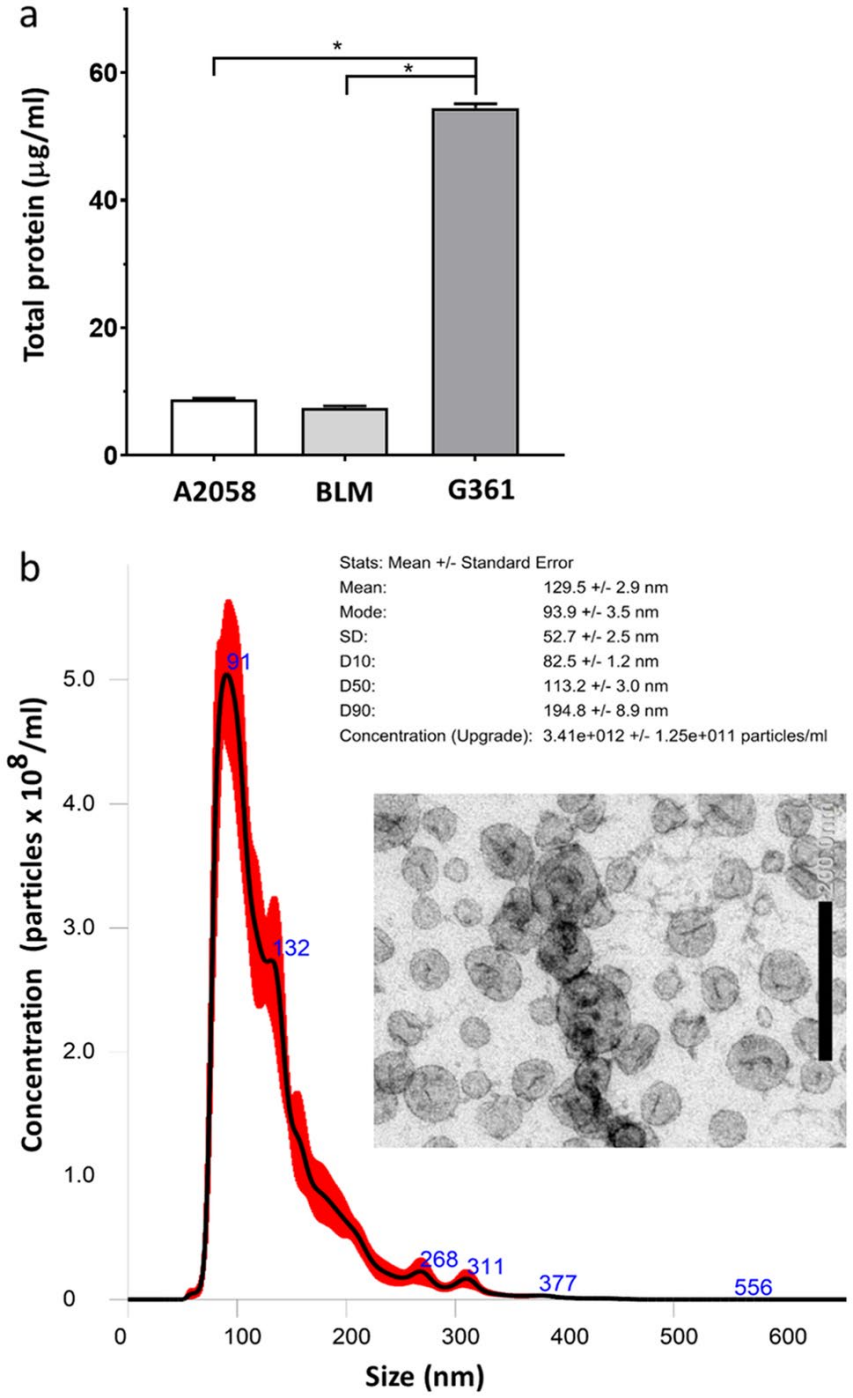
Characterisation of exosomes according to total protein detection, size and electron microscopy. **a** The graph shows that only G361 cells produced a sufficient quantity of exosomes; data represent quantification of three independent isolation experiments with A2058, BLM and G361 melanoma cell lines, respectively; error bars represent standard deviations of observed values; statistical significance calculated by non-parametric Tukey’s honest significance test (*p* value ≤ 0.05 was regarded as statistically significant). **b** Exosomes produced by G361 cells were homogenous concerning their size and morphology. Inset represents electron microscopic image presenting purified extracellular vesicles of usual exosomal size and morphology

Using cushioned-density gradient ultracentrifugation, we achieved high-quality exosome purification (Fig. 1). This procedure resulted in elimination of non-exosomal vesicles and also protein contaminants (Supplementary Figure 1), meaning around 90% of total proteins (Li et al. 2018). The exosome production was studied in three malignant melanoma cell lines: A2058, BLM and G361. Surprisingly, only the G361 melanoma line cells produced exosomes in amounts sufficient for further analysis and subsequent experiments (Fig. 1a). Therefore, G361-derived exosomes were characterised and complied with the size distribution and morphological criteria typical of exosomes by NTA and electron microscopy (Fig. 1b). Exosomes were dominantly present in density gradient fractions nos. 6–8 (Supplementary Figure 1a) having an expected exosome density of 1.08–1.13 g/cm^3^ (Brennan et al. 2020). All exosomes from studied cell lines exhibited surface protein markers CD9, CD63 and CD81 (Supplementary Figure 1b). Based on these observations, we confirmed that vesicles produced by G361 meet the criteria for being recognised as exosomes, and thus, we employed them for further experiments with HDFs and mCAFs. The quantity of exosomes produced by G361 is also followed proportionally by increased total protein concentration (Supplementary Figure 1c). We have not detected any significant residual contamination in commercially exosome-depleted serum (Supplementary Fig. 1a, lane 1). Therefore, we conclude that the observed effects can be attributed to G361-produced exosomes in our models.

### Effect of G361-derived exosomes on HDF and mCAF adhesion, proliferation and velocity in 2D and 3D

The RTCA iCELLigence™ instrument uses non-invasive electrical impedance monitoring to quantify attachment quality in a label-free, real-time manner. As fibroblast seeding density was identical, the measured values reflect the physical interaction of adherent cells with the surface of the electrode, primarily via surface receptors.

**Fig. 2:**
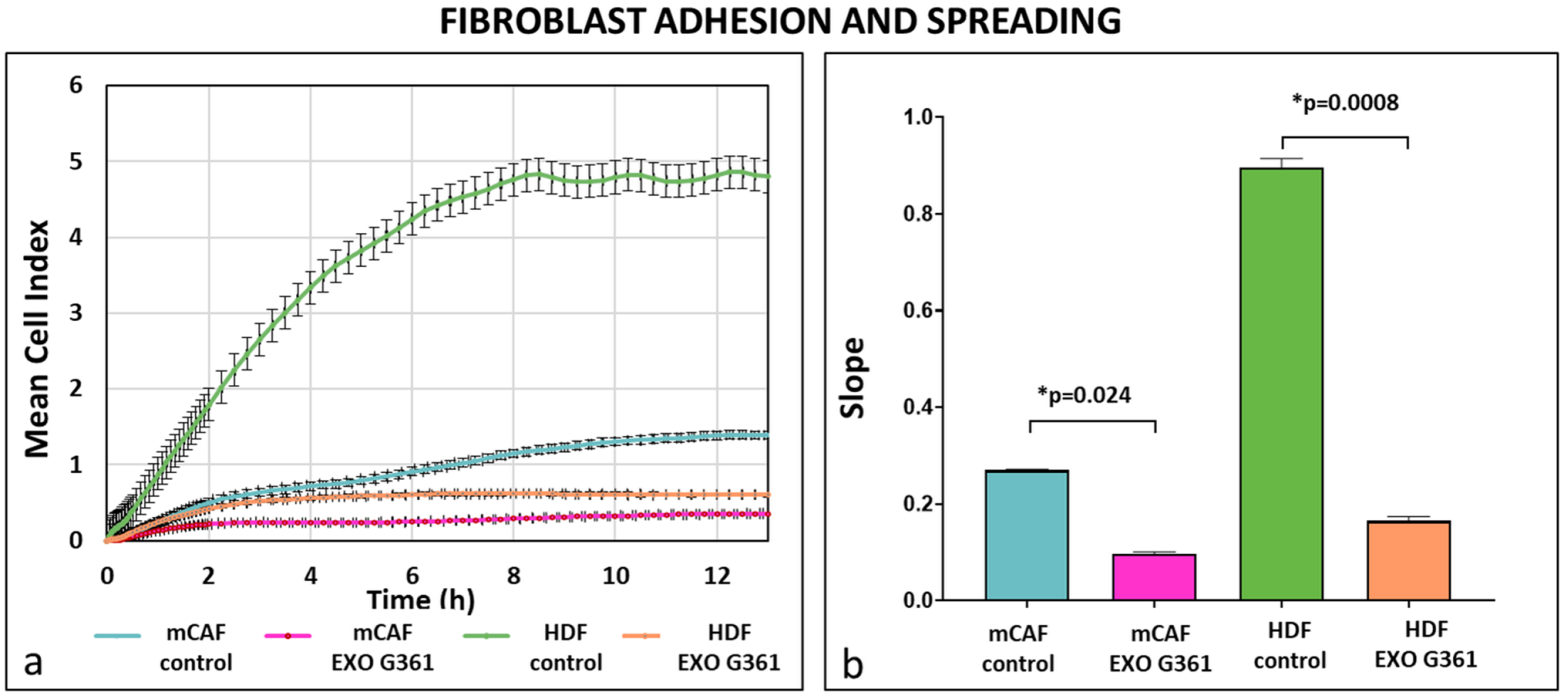
Exosomes decrease cell adhesion and spreading. **a** The graph shows cell adhesion as a mean cell index. Time point 0 represents the point of cell addition onto the xCELLigence plate. Before that, the cells were treated for 4 h with melanoma exosomes or with the medium without exosomes. **b** The rate of cell growth was determined by calculating the slope of the line between two given time points using xCelligence software. *p* values indicate statistical significance calculated by a two-tailed Student’s *t* test

Generally, the adhesive properties of mCAFs were lower than those of HDFs. In both fibroblast types, the addition of exosomes flattened the adhesion curves, suggesting decreased cell adhesion and spreading. This effect was more prominent in HDFs than in mCAFs, and the difference was statistically significant at the endpoint (Fig. 2). Moreover, the exosome-treated cells did not reach the maximum cell indices of control cells before reaching the curve plateau stage. This suggested that the lower adhesion of exosome-treated cells is not only a temporary post-seeding issue, but is persistent and cannot be rescued by subsequent exchange of media without exosomes (data not shown).

**Fig. 3:**
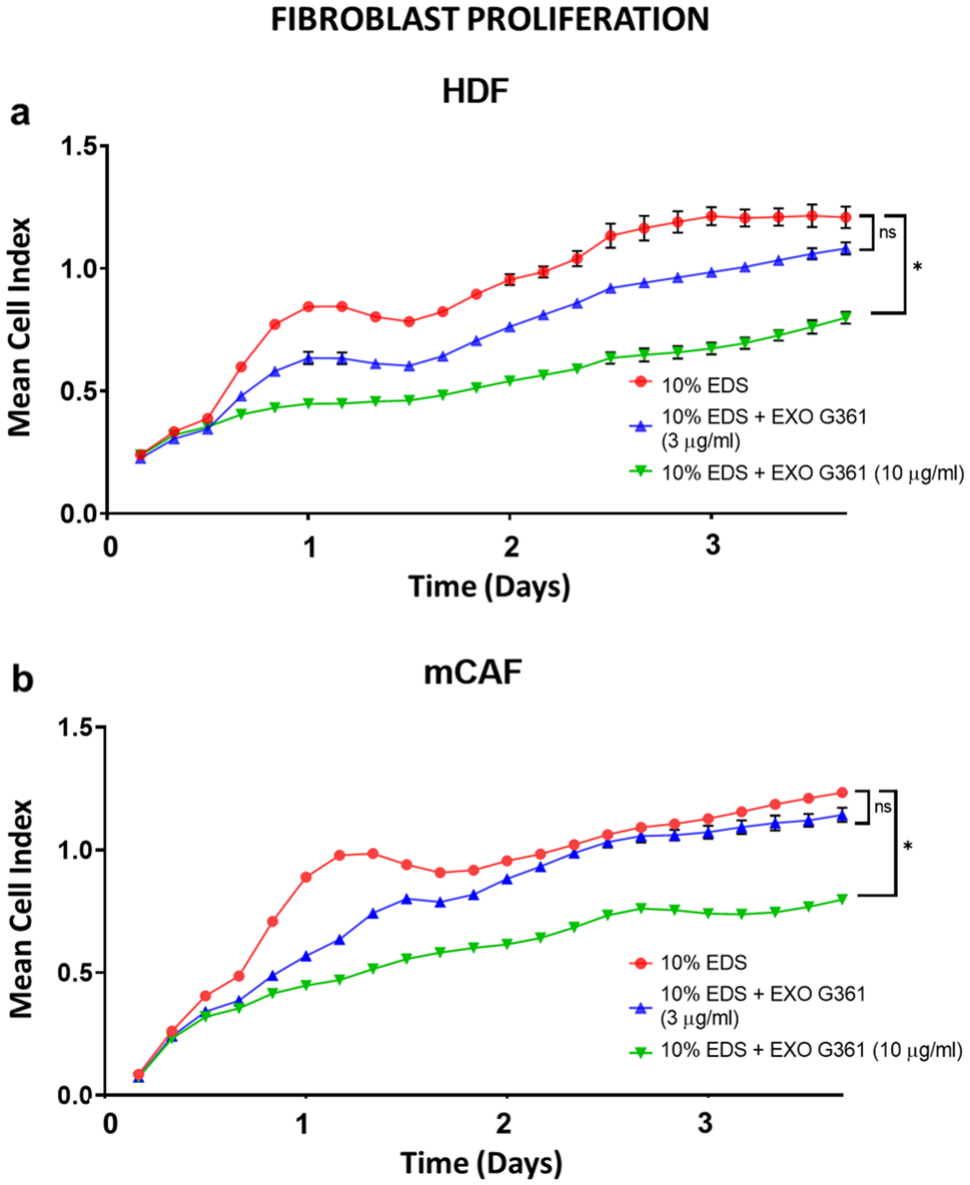
Exosomes decrease fibroblast proliferation. **a** The graph shows cell proliferation (90 h) of HDF as a mean cell index. **b** The graph shows mCAF proliferation (90 h) as a mean cell index. Time point 0 represents the point of cell addition onto the iCELLigence plate with exosomes (3 μg/ml blue, 10 μg/ml green) or without exosomes in DMEM + 10% EDS (red). Statistical significance (at the endpoint) was tested by Dunn’s post hoc multiple comparison test with Bonferroni correction (significant comparison with *p* value < 0.05 indicated by asterisk)

Once the cells are adjusted to their new environment, their proliferation can start again. The proliferation activity of fibroblasts is usually low within the initial 24 h after subculture. Starting day 2, changes of electrical impedance can reflect the increasing number of cells in culture wells. Similarly to the above-mentioned, the extent of proliferation of fibroblasts treated by exosomes was lower than that in the non-treated controls. This phenomenon was dependent on the concentration of applied exosomes (Fig. 3). Based on that, we decided to use a concentration of 10 µg/ml of exosomes as a reference in our experiments.

**Fig. 4:**
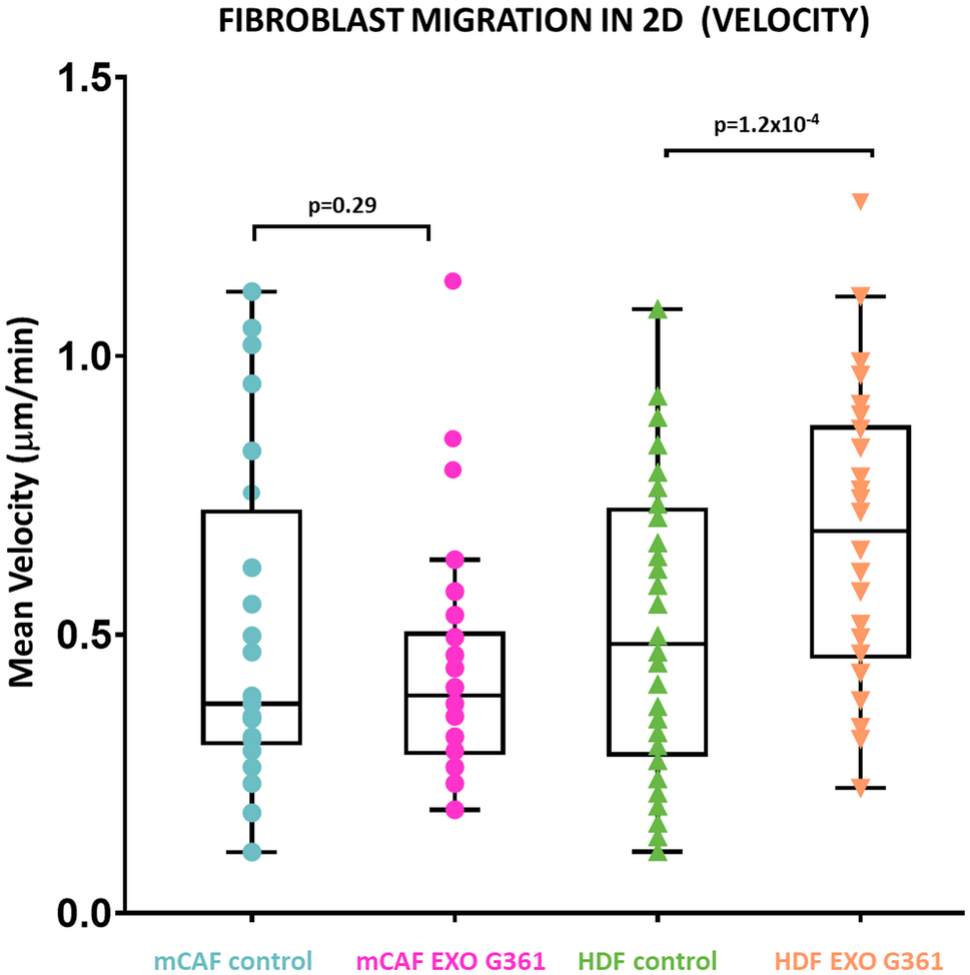
Exosomes increase the speed of cell migration in 2D. The box plot graph shows the cell speed differences between untreated and exosome-treated cells. *p* values indicate statistical significance calculated by a two-tailed Student’s *t* test

**Fig. 5:**
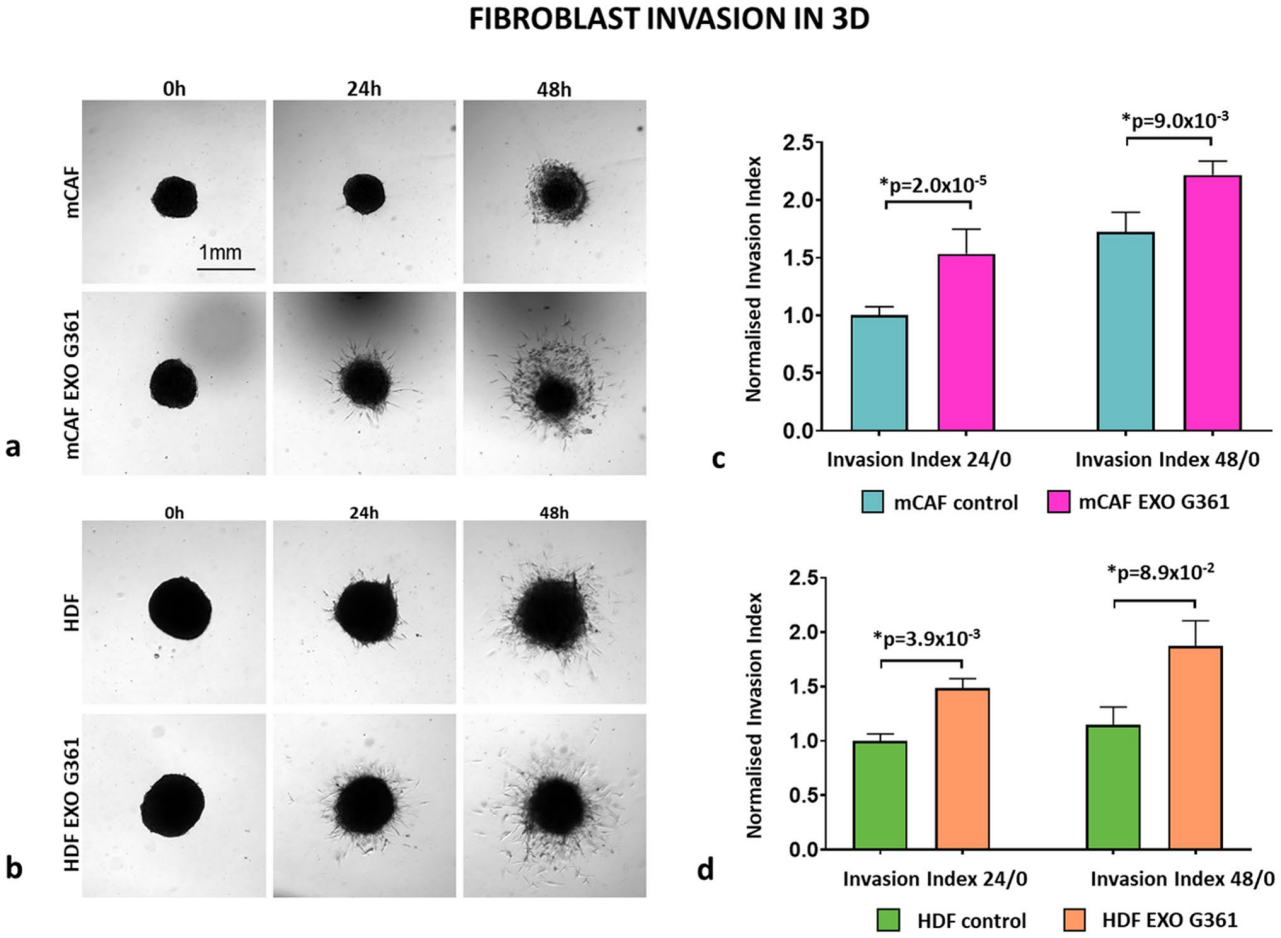
Exosomes enhance cell invasion in 3D. **a** 3D fibroblast spheroids formed of mCAF cells were treated with or without exosomes and imaged at time points 0, 24 and 48 h. The bar indicates 1 mm. **b** 3D fibroblast spheroids formed of HDF cells were treated with or without exosomes and imaged at time points 0, 24 and 48 h. **c** Bar graphs show the mean invasion index of mCAF cells at time points 24 and 48 h. **d** Bar graphs show the mean invasion index of HDF cells at time points 24 and 48 h. The *p* values in (**c**) and (**d**) indicate statistical significance calculated by two-tailed Student’s *t* test

Evaluation of the effect of exosomes on the velocity of the migration of both types of fibroblasts demonstrated no significant effect on mCAFs. However, we observed a significant increase in the velocity of HDFs (Fig. 4). This observation in 2D can be somewhat extended by results observed in 3D. In the collagen gel, application of G361-derived exosomes significantly increased the normalised invasion index of both types of fibroblasts, i.e., mCAFs and HDFs, in the 3D collagen gel (Fig. 5).

### Effect of exosomes on the invasion from composite melanoma spheroids in 3D collagen gels

**Fig. 6:**
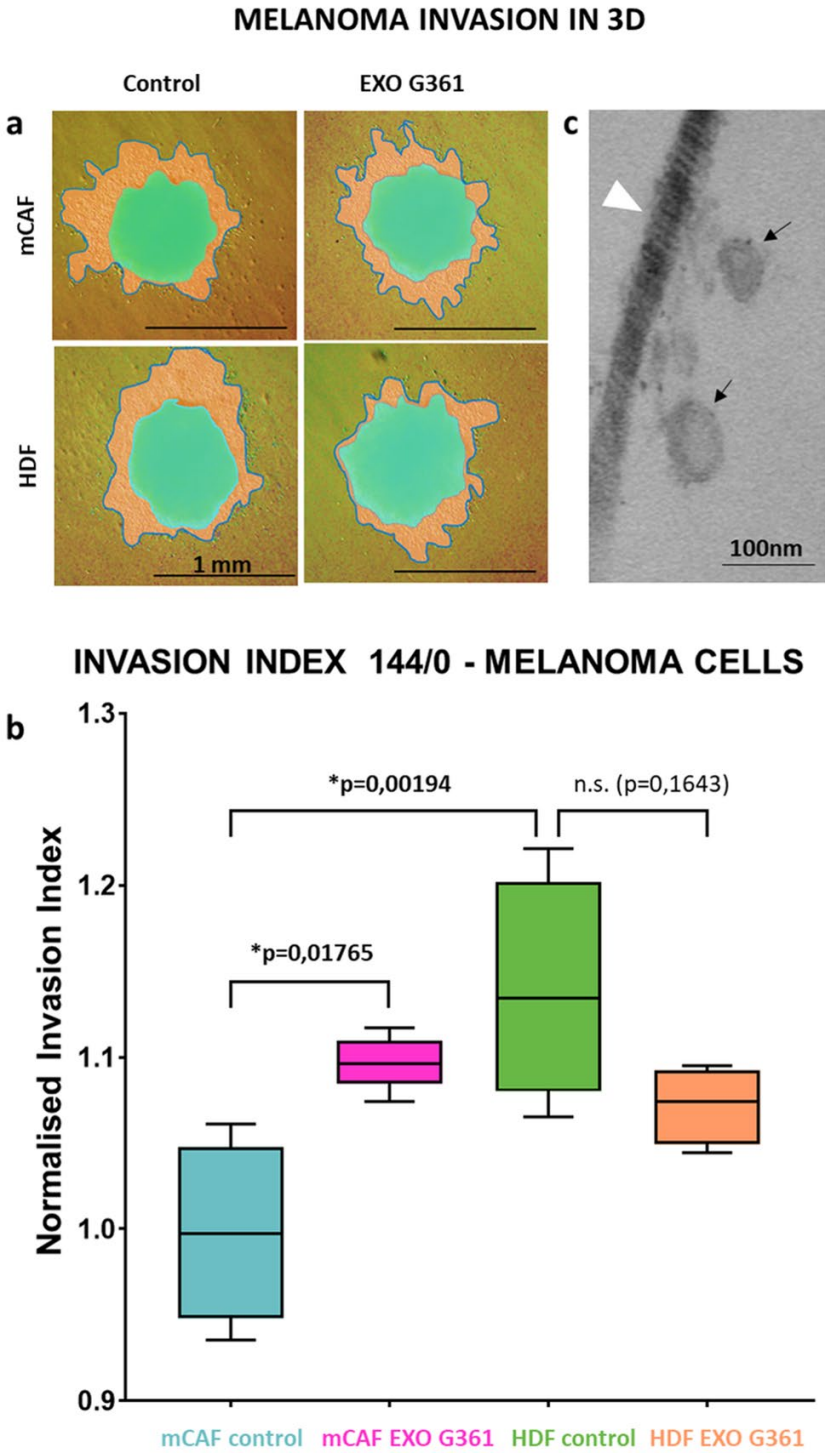
Exosomes enhance melanoma cell invasion in 3D. **a** 3D mixed spheroids formed of G-361 melanoma cells with fibroblasts (mCAFs and HDFs, respectively) were embedded in 3D collagen gel with or without exosomes and imaged at time points 0 h (green mask) and 144 h (orange mask with outline). **b** Box and whisker graphs show the mean invasion of G-361 cells at time point 144 h. **c** Electron microscopy of collagen gel with embedded exosomes (black arrow) and collagen fibril (white arrowhead). The bar in (**a**) indicates 1 mm and in (**c**) 100 nm. The *p* values in (**b**) indicate statistical significance calculated by non-parametric Tukey’s honest significance test (*p* value ≤ 0.05 was regarded as statistically significant)

**Fig. 7:**
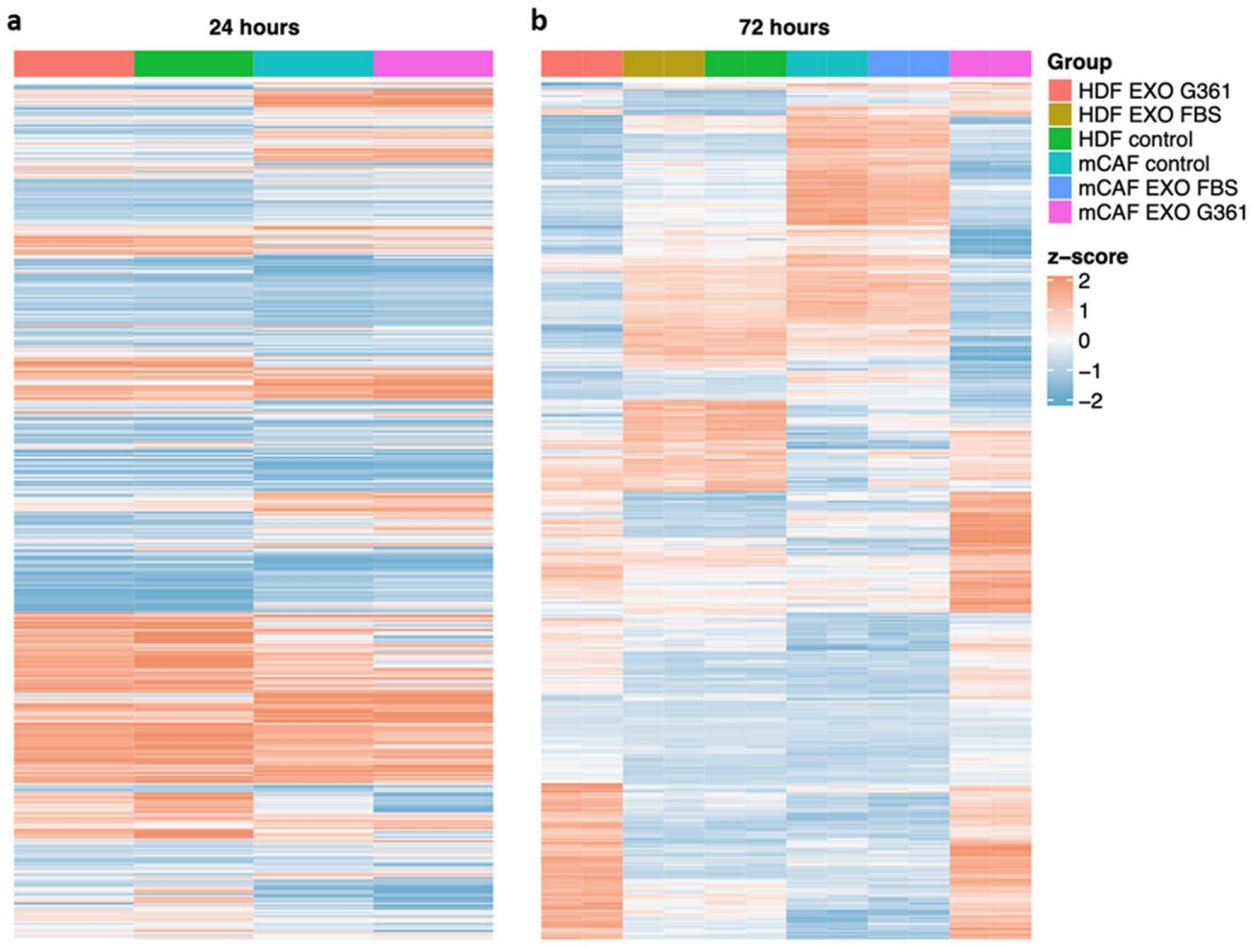
G-361-derived exosomes influenced the fibroblast transcriptome. **a** The heatmap presents the transcriptomic profile of HDFs and mCAFs 24 h after exosome stimulation. **b** The heatmap presents the transcriptomic profile of HDFs and mCAFs 72 h after exosome stimulation

For a long-term experiment maintained for 1 week, we mixed G361 melanoma cells with HDFs and mCAFs, respectively, to form heterogeneous spheroids as a more reliable 3D model of melanoma (Fig. 6a). These spheroids invaded collagen (Fig. 6b) and remained viable for 1 week (MTT test-based images not presented). To mimic the exosome-enriched microenvironment, the spheroids were embedded into collagen gels containing G361-derived exosomes (Fig. 6c). Indeed, the collagen gel casting procedure did not adversely impact the exosomes (Fig. 6c, arrow). Both cell types, i.e., melanoma cells as well as fibroblasts, migrated readily from composite spheroids (highlighted in green) to the collagen gel. These two invading cell types could be easily distinguished based on their cytological characteristics (Fig. 7a). Fibroblasts formed sparse outgrowths of predominantly individual cells (similar as presented in Fig. 5). Melanoma cells migrated mostly in densely packed outgrowths (highlighted in orange). G361-derived exosomes lowered invasion of melanoma cells from HDF-containing spheroids. Reversely, exosomes facilitated melanoma cell migration in the case of mCAF-containing spheroids. However intriguing, these differences were not statistically significant. Of note, we also observed a statistically significant difference between the extent of invasion from mCAF- and HDF-containing spheroids, respectively.

### Influence of exosomes on the HDF and mCAF transcriptome

**Fig. 8:**
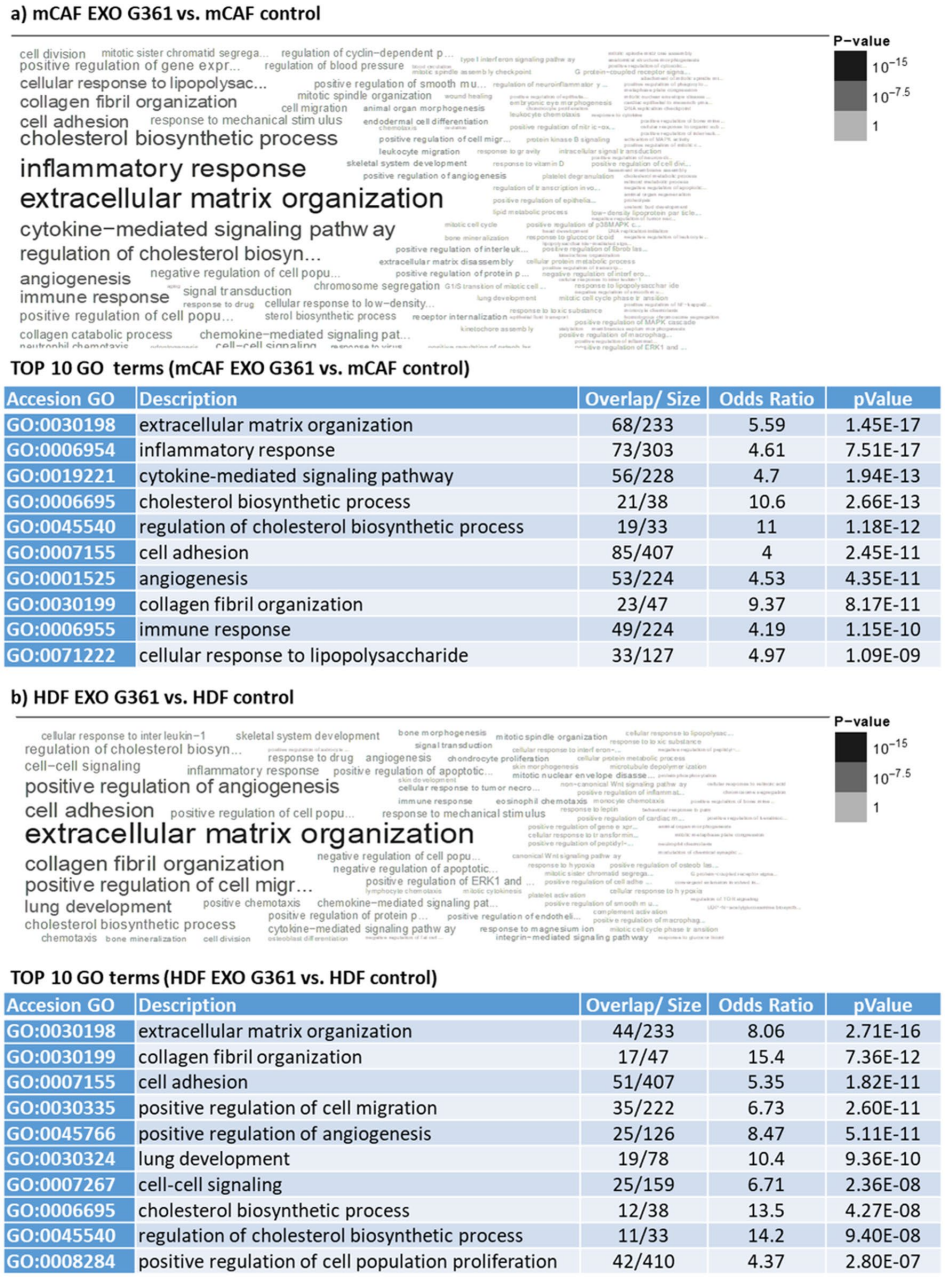
Comparison of the influence of G-361-derived exosomes on HDFs and mCAFs. The results of gene set enrichment analysis indicate that the G-361-derived exosomes influence extracellular matrix organisation in mCAFs (**a**) and HDFs (**b**). The exosomes also influence the inflammatory response and cytokine-mediated signalling pathway in mCAFs (**a**). The top ten GO terms for **a** and **b** are presented in tables below (based on *p* value)

In our previous work (Novotný et al. 2020), we observed three distinct subpopulations in otherwise homogeneous cultures of fibroblasts (HDFs and mCAFs) exposed to melanoma cells in a 3D model. Therefore, we have decided to use single-cell RNA sequencing to study potentially diverse effects of exosomes on different fibroblast subpopulations of HDFs and mCAFs. Surprisingly, the effect of G361 exosomes on the transcriptome of HDFs and mCAFs was not rapid, and we observed only modest changes at 24 h after stimulation by exosomes (Fig. 7a). However, 72 h after exosome application, notable changes in gene expression were observed in fibroblasts (Fig. 8b). The results were also compared with application of exosomes isolated from the normal foetal bovine serum, which resulted in a weaker effect in both HDFs and mCAFs (Supplementary Figure 2).

In transcriptomic analysis, we have primarily focused on three important aspects potentially related to our above-mentioned findings. We have summarised in Supplementary Table 2 differentially regulated genes in KEGG pathways for (a) focal adhesion, (b) cell migration and (c) cell proliferation. We have highlighted genes which were significantly deregulated in both HDF and mCAF fibroblasts.

Exosomes influenced organisation of extracellular matrix in both types of fibroblasts. We have observed discordant deregulation of tenascin C (*TNC*) where mCAF increased expression after exosome stimulation. Similar effect was observed for integrin alpha-8 (*ITGA8*). We have also observed a significant increase of podoplanin (*PDPN*) in mCAFs.

In contrast to HDFs, exosomes significantly influenced the inflammatory response and cytokine-mediated signalling pathways and cholesterol synthesis in mCAFs (Fig. 8a, b). Exosomes also significantly stimulated mevalonate pathways in CAFs (Supplementary Figure 3).

**Fig. 9:**
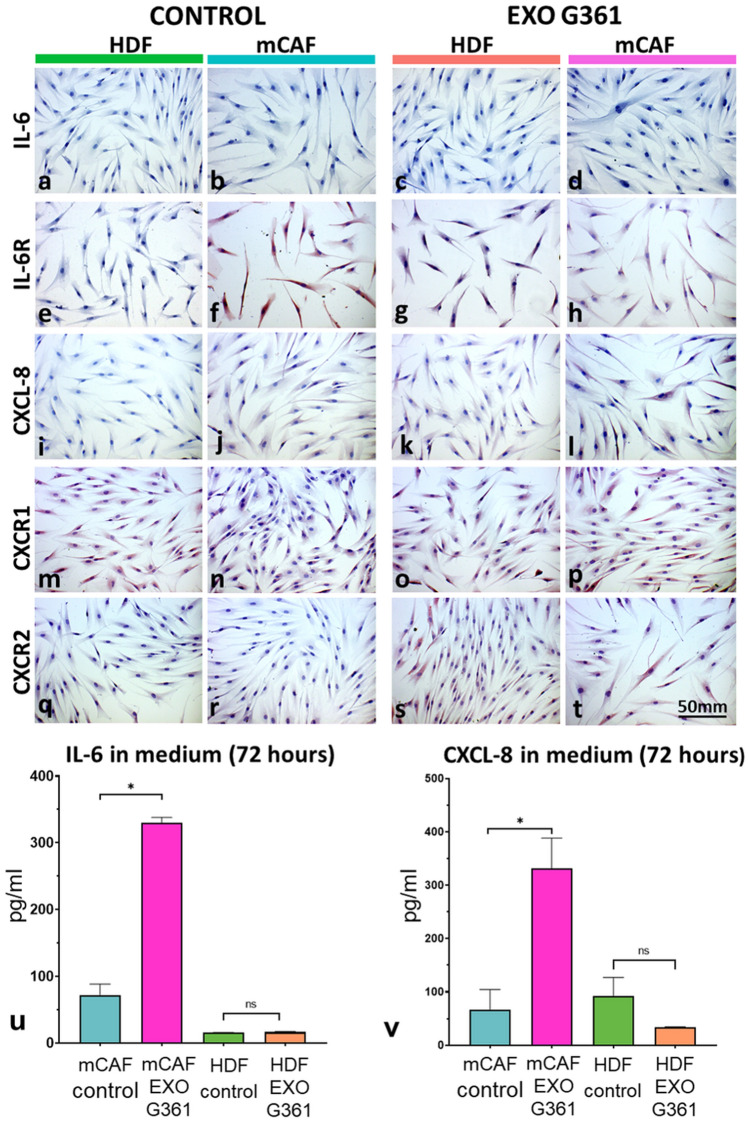
Immunocytochemical analysis of HDFs and mCAFs treated for 24 h with exosomes in culture medium. **a**–**d** IL-6 was not detected in cells; **e**–**h** low–medium signal of IL-6R in fibroblasts; **i**–**l**) detection of CXCL-8 (negative); **m**–**p**: detection of CXCR1 (low–negative); **q**–**t** CXCR2 (low–negative). Bar represents 50 μm. **u** IL-6 was detected in conditioned media after 72 h (pg/ml) by ELISA. **v** CXCL-8 was detected in conditioned media after 72 h (pg/ml) by ELISA. Error bars represent standard deviation (two independent experiments, three technical replicates for each. *p* values indicate statistical significance calculated by a two-tailed Student’s *t* test

Previously, we have demonstrated (Novotný et al. 2020) that cancer-associated fibroblasts differ from normal cells by excessive expression of IL-6, CXCL-8 and their receptors. Upon this ground, we focused our interest on these proteins in the experiment of 24-h exosome exposure to HDFs and mCAFs. Employing immunocytochemistry, we detected no presence of IL-6 in all cultured fibroblasts before and after G361 exosome treatment (Fig. 9a–d). A low but specific signal of IL-6R was present in cultured cells except for non-treated HDFs (Fig. 9e–h). Only insignificant differences were observed in the IL-6R gene on a mRNA level (not shown). CXCL-8 was not detected (Fig. 9i–l). Both receptors CXCR1 and CXCR2 (Fig. 9m–t) were low to negative in all cultured fibroblasts. This is in good agreement with the absence of IL-6, CXCL-8, CXCR1 and CXCR2 at the mRNA level before and after G361 exosome treatment.

**Fig. 10:**
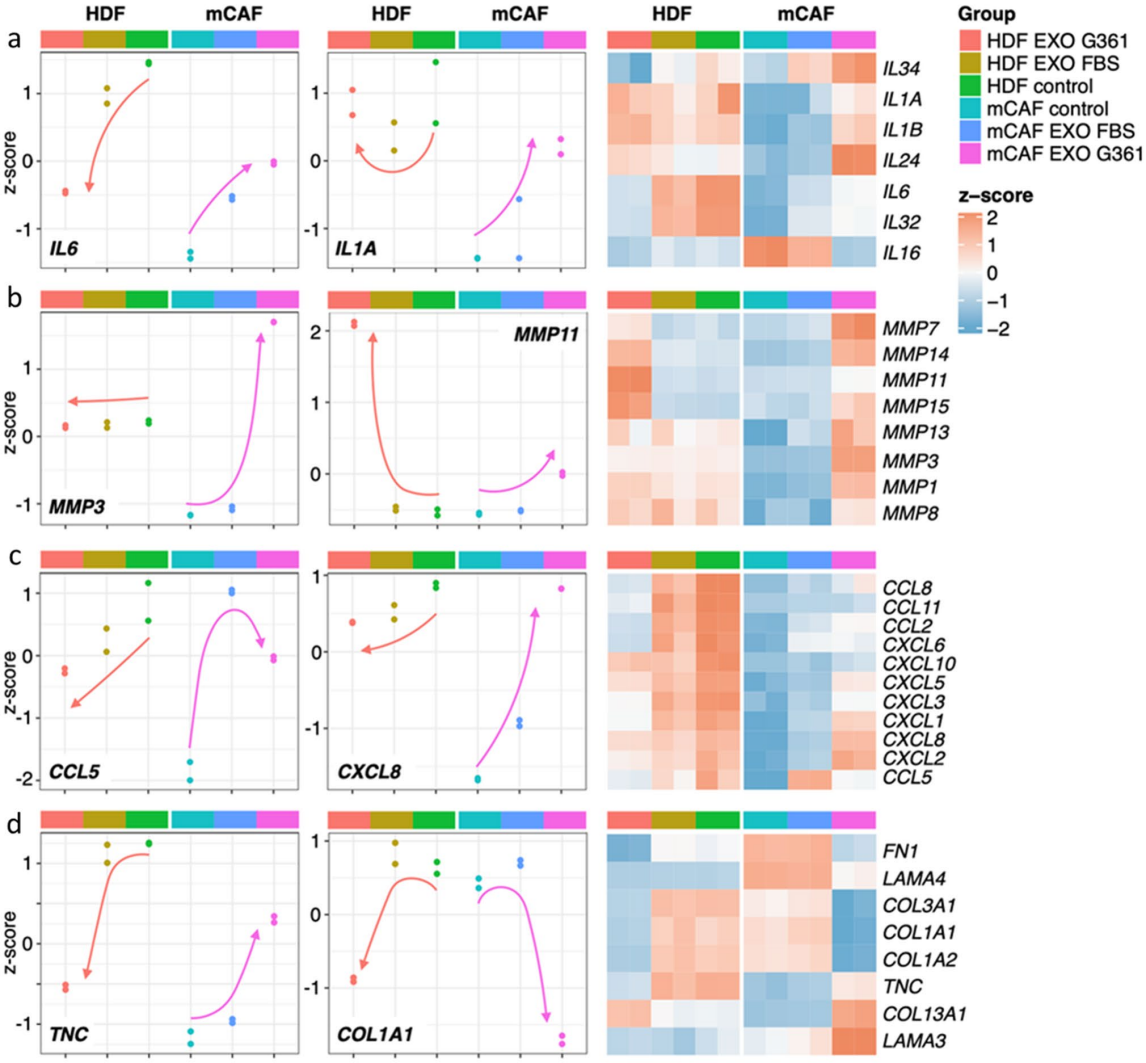
Transcriptional changes in HDFs and mCAFs after 72 h of exosomal stimulation (G361-derived vs FBS-derived exosomes). Panel **a** presents global changes with emphasis on interleukins, **b** represents matrix metalloproteinases, **c** represents chemokines and **d** represents extracellular matrix molecules. The overall trend is indicated by the orange (HDFs) or purple arrow (mCAFs)

Our further interest was focused on detailed analysis of the influence of both types of exosomes, i.e., from foetal bovine serum and G361 melanoma cells, on both HDFs and mCAFs after 72 h of treatment. We present here representatives of interleukins (Fig. 10a), matrix metalloproteinases (Fig. 10b), chemokines (Fig. 10c) and extracellular matrix molecules (Fig. 10d). Interestingly, the effect of exosomes on the activity of some genes such as *IL1A, IL6, MMP3*, *CCL5*, *CXCL8* or *TNC* is different in HDFs and mCAFs. On the other hand, it is similar in the case of *MMP11* and *COL1A1*. To support the validity of these mRNA profiles, we have tested protein levels of IL-6 and CXCL8 released in fibroblasts -conditioned media by ELISA (Fig. 9u–v). We observed initially high IL-6 in mCAFs-conditioned media and further increase upon exosome stimulation. The IL-6 level in HDF control was initially lower, and differences induced by exosomes were subtle. In mCAFs, CXCL8 followed a similar trend. Of note, we also detected the effect of exosomes isolated from FBS, but these were not so potent as in the case of G361-derived exosomes (Fig. 10a–d).

**Fig. 11:**
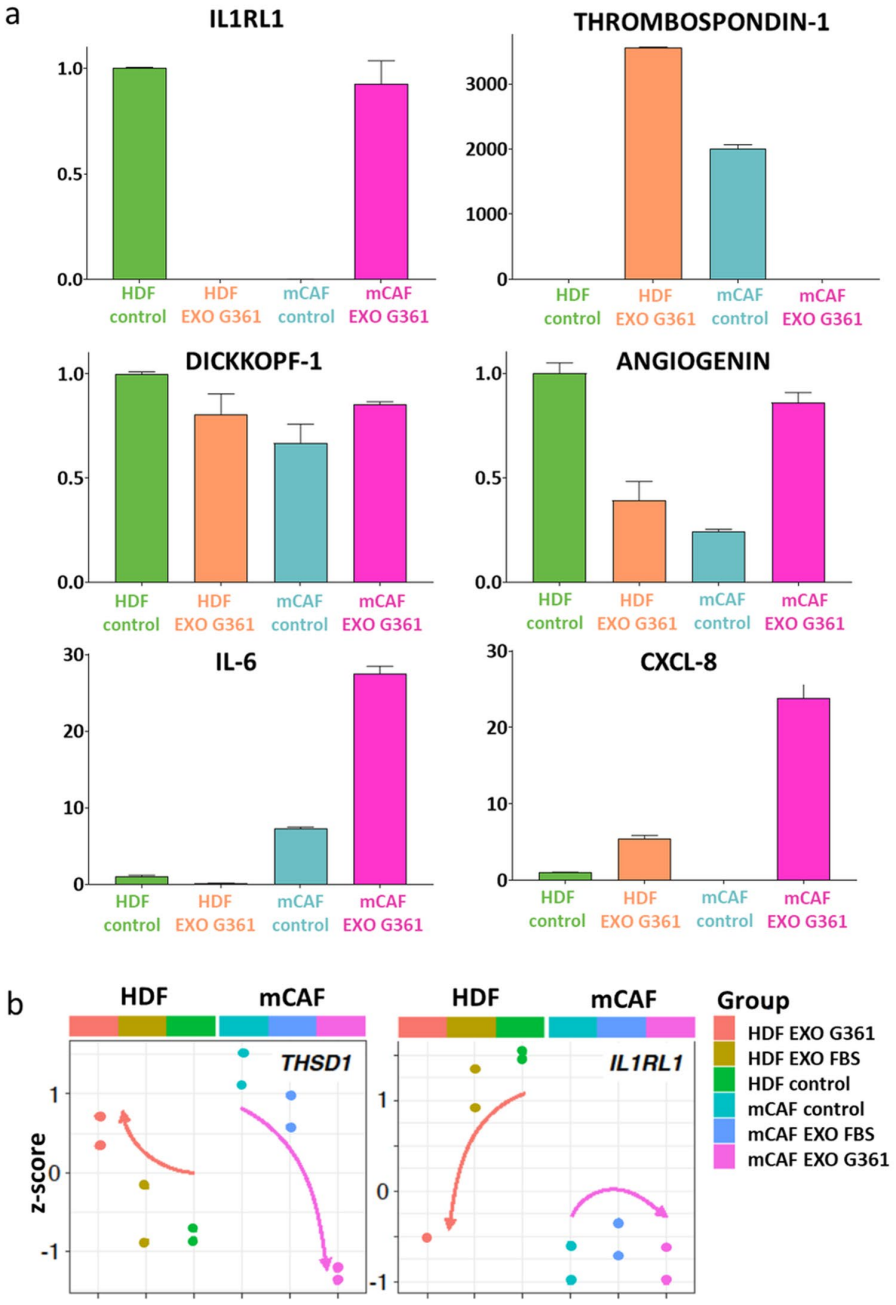
Panel **a** presents bar graphs representing changes in selected proteins in fibroblast-conditioned media after 72 h of stimulation by G-361 exosomes (detected in the Proteome Profiler Human XL Cytokine Array). Data represent an average of two capture spots for a particular analyte, and for purposes of comparison were normalised to the HDF level; error bar represents standard deviation. **b** The expression of both IL1R1 and thrombospondin-1 was also compared at the mRNA level, and a very similar trend was shown in the activity of *THSD1* gene for both types of fibroblasts and in the case of *1L1RL1* gene for HDFs. The overall trend is indicated by the orange (HDFs) or purple arrow (mCAFs)

Using the Proteome Profiler XL kit, we analysed supernatants prepared from fibroblast cell cultures with or without G361-derived exosomes for secretion of 105 cytokines. Selected data are presented in a bar graph (Fig. 11a). The IL1RL1 protein was present only in the non-treated HDFs and G361 exosome-treated mCAFs. On the other hand, thrombospondin-1 was present in the G361 exosome-treated HDFs and non-treated mCAFs. Dickkopf-1 was observed in both types of fibroblasts, and it was downregulated by exosomes in HDFs and upregulated by application of exosomes in mCAFs. The same and pronounced trend was also observed in the expression of angiogenin. Application of exosomes strongly increased the presence of IL-6 and CXCL-8, especially in mCAFs (Fig. 11a). The expression of both IL1R1 and thrombospondin-1 was also compared at the mRNA level. We observed a very similar trend in the activity of the *THSD1* gene for both types of fibroblasts, corresponding well to proteomic results. There was a partial similarity in the case of *1L1RL1* gene in HDFs (Fig. 11b).

## Discussion

We isolated extracellular vesicles produced by melanoma cells, which meet the criteria of exosomes (Théry et al. 2006; Xu et al. 2015). It was observed earlier that melanoma cell lines produce exosomes (Lazar et al. 2015); however, their cargo and function may vary significantly. In our experiments, only one melanoma cell line, G361, produced exosomes in sufficient quantity, despite its lower migratory and invasive capability in comparison to cell line A2058 (Kim et al. 2017).

Of note, G361 was established from a primary malignant melanoma of a 31-year-old male Caucasian (Peebles et al. 1978). G-361 thus represents a model potentially more sensitive to microenvironmental cues (Preisner et al. 2018) than other commonly used melanoma cell lines, which frequently originate from metastatic lesions. It was hypothesised (Hsu et al. 2000) that loss of microenvironmental dominance over early melanoma cells correlates with downregulation of adhesive molecules and results consequently in increased invasiveness of the tumour. In contrast, cells from metastatic lesions are highly invasive and already less sensitive to the influence of the microenvironment. Exosomes can represent such microenvironmental stimuli.

Exosomes secreted by malignant cells are an integral part of the tumour microenvironment. They can interact with collagen fibres, the most abundant ECM molecules in the dermis, and likely with numerous other ECM molecules. It was hypothesised earlier by others (Vlodavsky et al. 1990) that ECM provides a storage depot for biologically active agents that are stabilised, protected and gradually released. The exosome cargo can provide a persistent mode of action compared to the same molecules in a fluid phase. It can lead to stable tuning of the microenvironment facilitating cancer progression.

The interaction of exosomes with ECM was described in many cancer types and other pathological conditions (Buzás et al. 2018; Xu et al. 2019). In our experiments, G361-derived exosomes immobilised in high concentration to collagen non-significantly suppressed migration of cancer cells from spheroids containing normal fibroblasts. However, in the context of the highly permissive microenvironment represented here by mCAFs in composite spheroids, exosomes revealed an opposite trend.

Application of these exosomes to 2D culture of HDFs or mCAFs inhibited their adhesion and proliferation in a concentration-dependent manner. Similarly, umbilical cord stem cell-derived or oral epithelial cell-derived exosomes have an inhibitory effect on fibroblast proliferation (Sjöqvist et al. 2019; Yao et al. 2020). Regarding mobility, exosomes were not able to influence the velocity of mCAFs, but they stimulated the migration of HDFs. Ovarian- or prostate cancer-derived exosomes also reduce adhesion and stimulate the velocity of fibroblast migration (McAtee et al. 2019; Lee et al. 2020).

However, in the 3D system in collagen gel, we observed that exosomes positively influenced cell migration of both HDFs and mCAFs. These differences between 2D and 3D cultures are well known (Duval et al. 2017). We must highlight here that collagen gels do not represent a complex architecture of dermal extracellular matrix. This model can be in fact much closer to the stochastic arrangement observed during early wound healing. This migration-enhancing effect of exosomes seems to be particularly important, because fibroblasts can lead to tumour cell invasion (Liu et al. 2019). Inhibition of the motility of fibroblasts and their adhesion to tumour cells can be achieved by application of INFγ. Of note, fibroblasts modified by DNA present in exosomes from uveal melanoma acquire properties of cancer cells, and they proliferate more and migrate more readily after application of cancer-derived exosomes (Tsering et al. 2020). Our observations harmonise well with these findings and indicate broad activity of cancer cell-derived exosomes on both normal and cancer-associated fibroblasts. We must acknowledge here that this effect is not uniform and highlights the functional diversity of fibroblasts under normal (Rodemann and Rennekampff 2011) and pathological (Bu et al. 2020) conditions. Such diversity can be documented by genes deregulated discordantly in comparison of HDF and mCAF after stimulation (Supplementary Table 2).

As fibroblast seeding density was identical in RTCA experiments, the measured values reflect the physical interaction of adherent cells with the surface of the electrode, primarily via surface receptors like integrins. Various cells can significantly differ in the types and numbers of these surface receptors. We have observed changes in expression of various integrins (*ITGA2*, *ITGA7*, *ITGA10*) in both fibroblast types in the same direction. Of note, *ITGA8* was deregulated discordantly and was increased in mCAFs in response to exosome stimulation. Identical trend was observed for *TNC*—it is known that this integrin is the receptor for tenascin.

The physical adherence of a cell can also be significantly modified by the environment and surface physiochemical features, e.g., substrate stiffness. Due to these factors, even cells of equal physical volume can spread to different extents and thus occupy an area of unequal size. There is no generally accepted precise timing that would allow us to separate the initial cell adhesion from consecutive cell spreading to achieve optimal surface attachment. This can also lad to differences in activity of focal-adhesin kinase (FAK) signalling pathways. One of these discordantly deregulated genes is *PDPN*. *PDPN* is known for induction of major reorganisation of the actin cytoskeleton, increased motility and decreased cell adhesion in tumours (Martín-Villar et al. 2005).

Histologic observations of desmoplastic reaction surrounding proliferating tumour buds were initially understood as a response of the host tissue against invasive cancer cells. It was taken as a thick tissue barrier against cancer invasion and potential metastasis. To initiate this fibroplasia, paracrine signalling across the tissue microenvironment is necessary, and it should facilitate alterations of cell proliferation and differentiation. Exosomes are an efficient tool for this information exchange across the niche. Indeed, HDFs in the G361 exosome-enriched environment slowed down melanoma cell invasion in our experiments. The naïve fibroblast population in real tissue, represented here by HDFs in spheroids, once stimulated by exosomes possibly acts as a defensive mechanism to insulate the tumour (Schäfer and Werner 2008). However, this is just a temporary advantage. In an extended time frame, the exosomes can also contribute to fibroblast recruitment and their transformation into myofibroblasts, e.g., by TFG-β-dependent mechanisms described in detail by others (Webber et al. 2010). It was suggested recently that α8β1 integrin enhances cellular contractility and TGFβ activity in liver fibrosis (Nishimichi et al. 2021). Similar can be expected in cancer. Hence, the defender can be easily corrupted and become a cancer abettor (Fiori et al. 2019).

G361-derived exosomes significantly influenced transcription profiles of both normal HDFs and mCAFs. Interestingly, the effect of exosomes observed on the HDF and mCAF transcriptomes was not the same. For example, the activity of genes for cytokines/chemokines such as *IL1*, *IL6*, *IL24*, *IL32*, *IL34*, *CCL2*, *CCL5*, *CCL8*, *CCL11*, *CXCL1*, *CXCL2*, *CXCL3*, *CXCL5*, *CXCL6*, *CXCL8* and *CXCL10* was upregulated in mCAFs and not affected/downregulated in HDFs. It suggests that mCAFs under exosomal stimuli shift the microenvironment to a chronic proinflammatory setting typical of cancer. On the other hand, the activity of genes for matrix metalloproteinases *MMP1*, *MMP3*, *MMP7*, *MMP8*, *MMP11*, *MMP13*, *MMP14* and *MMP15* was upregulated in mCAFs, and in the case of *MMP7*, *MMP11*, *MMP14* and *MMP15*, also in HDFs. It highlights the potential of the tumour microenvironment for dynamic structural changes allowing tumour invasion. Concerning the genes encoding production of extracellular matrix proteins, activation of genes for tenascin-C (*TNC*) or laminins (*LAM3, LAM4*) was observed after the application of exosomes to mCAFs. The activity of genes encoding collagen type 1 (*COL1A1*, *COL1A2*) and collagen type 3 (*COL3A1*) was reduced in both mCAFs and HDFs. These data have demonstrated that G361-derived exosomes enhance the remarkable properties of cancer-associated fibroblasts in mCAFs.

In closer detail, CAFs are not functionally uniform either. CAFs represent a heterogeneous population where distinct subpopulations differ in production of ECM and secretion of bioactive cytokines/chemokines/growth factors, as was demonstrated for example in melanoma, urothelial carcinoma or adenocarcinoma of the pancreas (Lin et al. 2020; Tsering et al. 2020; Novotný et al. 2020). Inflammation-supporting factors, predominantly IL-6 secreted by CAFs, enhance the aggressiveness of tumours, including cutaneous malignant melanoma. A biologically relevant increase in cancer cell proliferation and metastasis formation was observed in many types of malignant tumours due to the CAF activity (Lacina et al. 2019; Plzák et al. 2019; Kodet et al. 2020; Brábek et al. 2020). A similar tumour-promoting effect can be assigned to activation of the gene for ECM protein tenascin-C. This ECM protein is rich in the microenvironment of many types of tumours, including malignant cutaneous melanoma, where it seems to be associated with cancer cell survival and invasion (Grahovac et al. 2013; Shao et al. 2015).

The effect of exosomes on activation of matrix metalloproteinases also stimulated cancer cell invasiveness by degradation of ECM proteins. The exosome-activated mCAFs upregulate the activity of genes for MMP-1, MMP-2, MMP-7, MMP-8, MMP-13 and MMP-14 that are associated with cancer growth and progression. Part of them (MMP-1, MMP-2, MMP-7 and MMP-13) also participate in inflammatory processes under the control of IL-1, IL-6 and CXCL-8 that are upregulated in mCAFs treated with exosomes (Cabral-Pacheco et al. 2020). The functional link of MMP with IL-1, IL-6 and CXCL-8 in mCAFs is shown in Supplementary Figure 4. This orchestration supports the role of exosomes in inflammation and of its mediators in the progression of cutaneous malignant melanoma.

Two proteins, IL1RL1 and thrombospondin-1, are expressed differentially with or without exosome application in both HDFs and mCAFs. IL1RL1, the receptor for IL-33, is present in non-stimulated HDFs and exosome-stimulated mCAF. Its role in cancer progression is highly probable (Zhou et al. 2020). Thrombospondin is up/downregulated in different tumours (Wang et al. 2020). In cutaneous melanoma, thrombospondin seems to be a marker of poor prognosis stimulating tumour vascularisation (Trotter et al. 2003). The stimulation of angiogenin in mCAF after exosome application can also be associated with tumour vascularisation (Gorain et al. 2019). Dickkopf-1 is a Wnt inhibitor. It is strongly expressed in both HDFs and mCAFs. The interpretation of this finding is complicated because its role as tumour-promoting and tumour-inhibiting factor was described (Li et al. 2020). Since the knockdown of Dickkopf-1 has a suppressive role in apoptosis induced by the combination of temsirolimus and temozolomide in vitro, it should be hypothesised that its role in cutaneous melanoma is tumour suppressing (Niessner et al. 2017).

The upregulation of the mevalonate pathway in exosome-stimulated mCAFs may be associated with RHO-GTPases and actin cytoskeleton biology that may influence mCAF formation and function. It was suggested that in fibroblast-led collective invasion of tumour cells, leading and following cells differ in activity and roles of RhoGTPases (Gaggioli et al. 2007). The proposed employment of statins in the therapeutic manipulations of the tumour ecosystem harmonises with this observation (Emelyanova et al. 2019; Ji et al. 2020; Yu et al. 2021).

Based on these data, it can be concluded that exosomes derived from G361 melanoma cells have a stimulatory effect on mCAF prepared from human cutaneous melanoma. It might be understood that fibroblasts turn into CAFs in the niche of the tumour microenvironment by the process of gradual and slow recruitment. This paradigm can also be extended to other tumour-supporting populations, including macrophages (Gok Yavuz et al. 2019).

Exosomes produced by cancer cells contribute to the co-evolution of cancer cells with their microenvironment during cancer formation and propagation (Valcz et al. 2020). Hu and Hu observed that exosomes prepared from melanoma cells re-programme NIH/3T3 to CAFs (Hu and Hu 2019). However, it must be highlighted in this context that 3T3 cells were established from mouse embryonic tissue (Todaro and Geen 1963), and they greatly differ from normal adult dermal fibroblasts. In the broader view of the whole organism, melanoma-derived exosomes are released to body fluids and thus influence distant fibroblasts to develop a premetastatic microenvironment (la Shu et al. 2018). This positive systemic effect of melanoma cell-derived exosomes should be reflected by tumour biology research in the nearest future. Because CAFs are very important players in information transfer in the tumour microenvironment, their therapeutic targeting may be beneficial for patient therapy in the future.

## Supplementary Information

Below is the link to the electronic supplementary material.

Supplementary file 1 (TIF 5805 kb) Protein fractions separated from G361 conditioned media. Western blot detection of exosome surface markers. Comparison of G361-isolated exosome number and protein concentration trend: (a) G361-derived extracellular vesicles were separated in fractions 1–12 by gradient ultracentrifugation. Exosomes were present in fractions 6–8. These fractions were further analysed by western blotting. (b) Exosome markers CD9, CD63 and CD81 were studied in: lane 1, 5% Thermo Scientific exosome-depleted FBS-enriched DMEM culture media (no band present), lanes 2–4 represent 5% Thermo Scientific exosome-depleted FBS-enriched DMEM supplemented by G361 self-prepared exosomes from fractions 6, 7 and 8, respectively. Bands with expected MW of CD9, CD63 and CD81 were detected. (c) Comparison of quantification of exosomes isolated by cushioned-density gradient ultracentrifugation. The number of cells (for normalisation) was counted after media harvesting using a haemocytometer. Concentration of exosomes (particles/mL) isolated from three melanoma cell lines were analysed by NTA using the NanoSight NS300 instrument and normalised (blue columns). Protein content in isolated exosomes was determined by BCA assay for WB protein loading (grey columns, represented in μg per 100,000,000 cells). Every column represents data from three independent experiments; error bars represent observed standard deviations.

Supplementary file 2 (TIF 24273 kb) G-361-derived and to a lesser extent FBS-derived exosomes influenced the fibroblast transcriptome. The heatmap presents the transcriptomic profile of HDFs and mCAFs 72 hours after exosome stimulation.

Supplementary file 3 (TIF 15273 kb) G-361-derived exosomes stimulate the mevalonate synthesis pathway in the studied fibroblasts.

Supplementary file 4 (TIF 16372 kb) STRING database - Protein-Protein Interaction Networks Functional Enrichment Analysis. Functional link of MMPs with prominent proinflammatory molecules IL-1, IL-6 and CXCL-8 frequently expressed in mCAFs. STRING interaction network. https://string-db.org/cgi/network?taskId=bJAkTLRhTlxn&sessionId=bvZh9tqml4fP. Accessed 4 May 2021

Supplementary file 5 (TIF 4703 kb) Cell tracking experiments workflow. a) Scheme of image processing for single-cell tracking analysis using TrackMate plug-in of ImageJ/Fiji. The processing was done in three consecutive steps: image stitching at each time point (1), cell segmentation using background suppression, Edge Finder tool and binary masking (2), and cell tracking using TrackMate plug-in (yellow lines display cell tracks). b) Maximal velocity. c) Minimal velocity

Supplementary file 6 (DOCX 16 kb)

Supplementary file 7 (XLSX 25 kb)

## Data Availability

All transcriptomic data has been deposited in the ArrayExpress database (http://www.ebi.ac.uk/arrayexpress) under accessions E-MTAB-10278 and E-MTAB-10290.

## References

[CR1] Alberts B, Johnson A, Lewis J, et al (2002) Cancer as a Microevolutionary Process. Available from: https://www.ncbi.nlm.nih.gov/books/NBK26891/. Accessed 01 June 2021

[CR2] Ashburner M, Ball CA, Blake JA (2000). Gene ontology: tool for the unification of biology. Nat Genet.

[CR3] Brábek J, Jakubek M, Vellieux F (2020). Interleukin-6: molecule in the intersection of cancer, ageing and COVID-19. Int J Mol Sci.

[CR4] Brennan K, Martin K, FitzGerald SP (2020). A comparison of methods for the isolation and separation of extracellular vesicles from protein and lipid particles in human serum. Sci Rep.

[CR5] Brustugun OT, Møller B, Helland Å (2014). Years of life lost as a measure of cancer burden on a national level. Br J Cancer.

[CR6] Bu L, Baba H, Yasuda T (2020). Functional diversity of cancer-associated fibroblasts in modulating drug resistance. Cancer Sci.

[CR7] Buzás EI, Tóth E, Sódar BW, Szabó-Taylor K (2018). Molecular interactions at the surface of extracellular vesicles. Semin Immunopathol.

[CR8] Cabral-Pacheco GA, Garza-Veloz I, Castruita-De la Rosa C (2020). The roles of matrix metalloproteinases and their inhibitors in human diseases. Int J Mol Sci.

[CR9] Cavallari C, Camussi G, Brizzi MF (2020). Extracellular vesicles in the tumour microenvironment: eclectic supervisors. Int J Mol Sci.

[CR10] Duval K, Grover H, Han LH (2017). Modeling physiological events in 2D vs. 3D cell culture. Physiology.

[CR11] Dvořánková B, Lacina L, Smetana K, Turksen K (2019). Isolation of normal fibroblasts and their cancer-associated counterparts (CAFs) for biomedical research. Skin stem cells: methods and protocols.

[CR12] Egeblad M, Nakasone ES, Werb Z (2010). Tumors as organs: complex tissues that interface with the entire organism. Dev Cell.

[CR13] Emelyanova L, Sra A, Schmuck EG (2019). Impact of statins on cellular respiration and de-differentiation of myofibroblasts in human failing hearts. ESC Heart Fail.

[CR14] Ewels PA, Peltzer A, Fillinger S (2020). The nf-core framework for community-curated bioinformatics pipelines. Nat Biotechnol.

[CR15] Falcone I, Conciatori F, Bazzichetto C (2020). Tumor microenvironment: Implications in melanoma resistance to targeted therapy and immunotherapy. Cancers.

[CR16] Fiori ME, di Franco S, Villanova L (2019). Cancer-associated fibroblasts as abettors of tumor progression at the crossroads of EMT and therapy resistance. Mol Cancer.

[CR17] Gaggioli C, Hooper S, Hidalgo-Carcedo C (2007). Fibroblast-led collective invasion of carcinoma cells with differing roles for RhoGTPases in leading and following cells. Nat Cell Biol.

[CR18] Gener Lahav T, Adler O, Zait Y (2019). Melanoma-derived extracellular vesicles instigate proinflammatory signaling in the metastatic microenvironment. Int J Cancer.

[CR19] Gok Yavuz B, Gunaydin G, Gedik ME (2019). Cancer associated fibroblasts sculpt tumour microenvironment by recruiting monocytes and inducing immunosuppressive PD-1 + TAMs. Sci Rep.

[CR20] Gorain B, Choudhury H, Yee GS, Bhattamisra SK (2019). Adenosine receptors as novel targets for the treatment of various cancers. Curr Pharm Des.

[CR21] Grahovac J, Becker D, Wells A (2013). Melanoma cell invasiveness is promoted at least in part by the epidermal growth factor-like repeats of tenascin-C. J Invest Dermatol.

[CR22] Gu Z, Eils R, Schlesner M (2016). Complex heatmaps reveal patterns and correlations in multidimensional genomic data. Bioinformatics.

[CR23] Hamidi H, Lilja J, Ivaska J (2017). Using xCELLigence RTCA instrument to measure cell adhesion. Bio-Protocol.

[CR24] Hsu M-Y, Meier FE, Nesbit M (2000). E-cadherin expression in melanoma cells restores keratinocyte-mediated growth control and down-regulates expression of invasion-related adhesion receptors. Am J Pathol.

[CR25] Hu T, Hu J (2019). Melanoma-derived exosomes induce reprogramming fibroblasts into cancer-associated fibroblasts via Gm26809 delivery. Cell Cycle.

[CR26] Ji L, Liu C, Yuan Y (2020). Key roles of Rho GTPases, YAP, and Mutant P53 in anti-neoplastic effects of statins. Fundam Clin Pharmacol.

[CR27] Jobe NP, Rösel D, Dvořánková B (2016). Simultaneous blocking of IL-6 and IL-8 is sufficient to fully inhibit CAF-induced human melanoma cell invasiveness. Histochem Cell Biol.

[CR28] Jobe NP, Živicová V, Mifková A (2018). Fibroblasts potentiate melanoma cells in vitro invasiveness induced by UV-irradiated keratinocytes. Histochem Cell Biol.

[CR29] Kim D, Langmead B, Salzberg SL (2015). HISAT: a fast spliced aligner with low memory requirements. Nat Methods.

[CR30] Kim HY, Lee H, Kim SH (2017). Discovery of potential biomarkers in human melanoma cells with different metastatic potential by metabolic and lipidomic profiling. Sci Rep.

[CR31] Kodet O, Kučera J, Strnadová K (2020). Cutaneous melanoma dissemination is dependent on the malignant cell properties and factors of intercellular crosstalk in the cancer microenvironment (review). Int J Oncol.

[CR32] Kučera J, Strnadová K, Dvořánková B (2019). Serum proteomic analysis of melanoma patients with immunohistochemical profiling of primary melanomas and cultured cells: pilot study. Oncol Rep.

[CR33] la Shu S, Yang Y, Allen CL (2018). Metabolic reprogramming of stromal fibroblasts by melanoma exosome microRNA favours a pre-metastatic microenvironment. Sci Rep.

[CR34] la Shu S, Matsuzaki J, Want MY (2020). An immunosuppressive effect of melanoma-derived exosomes on NY-ESO-1 antigen-specific human CD8+ T cells is dependent on IL-10 and independent of BRAFV600E mutation in melanoma cell lines. Immunol Investig.

[CR35] Lacina L, Plzak J, Kodet O (2015). Cancer microenvironment: what can we learn from the stem cell niche. Int J Mol Sci.

[CR36] Lacina L, Brábek J, Král V (2019). Interleukin-6: a molecule with complex biological impact in cancer. Histol Histopathol.

[CR37] Lazar I, Clement E, Ducoux-Petit M (2015). Proteome characterization of melanoma exosomes reveals a specific signature for metastatic cell lines. Pigment Cell Melanoma Res.

[CR38] Lee AH, Ghosh D, Quach N (2020). Ovarian cancer exosomes trigger differential biophysical response in tumor-derived fibroblasts. Sci Rep.

[CR39] Li K, Wong DK, Hong KY, Raffai RL (2018). Cushioned-density gradient ultracentrifugation (C-DGUC): a refined and high performance method for the, isolation, characterization, and use of exosomes. Methods Mol Biol.

[CR40] Li J, Gao Y, Yue W (2020). The clinical diagnostic and prognostic value of dickkopf-1 in cancer. Cancer Manag Res.

[CR41] Liao Y, Smyth GK, Shi W (2014). featureCounts: an efficient general purpose program for assigning sequence reads to genomic features. Bioinformatics.

[CR42] Lin W, Noel P, Borazanci EH (2020). Single-cell transcriptome analysis of tumor and stromal compartments of pancreatic ductal adenocarcinoma primary tumors and metastatic lesions. Genome Med.

[CR43] Liu X, Zhu L, Wang R (2019). IFNγ inhibits fibroblast-leading tumor cell invasion through downregulating N-cadherin. Biochem Biophys Res Commun.

[CR44] Love MI, Huber W, Anders S (2014). Moderated estimation of fold change and dispersion for RNA-seq data with DESeq2. Genome Biol.

[CR45] Lun ATL, Riesenfeld S, Andrews T (2018). Distinguishing cells from empty droplets in droplet-based single-cell RNA sequencing data. bioRxiv.

[CR46] Luo W, Brouwer C (2013). Pathview: an R/Bioconductor package for pathway-based data integration and visualization. Bioinformatics.

[CR47] Martín-Villar E, Scholl FG, Gamallo C (2005). Characterization of human PA2.26 antigen (T1α-2, podoplanin), a small membrane mucin induced in oral squamous cell carcinomas. Int J Cancer.

[CR48] McAtee CO, Booth C, Elowsky C (2019). Prostate tumor cell exosomes containing hyaluronidase Hyal1 stimulate prostate stromal cell motility by engagement of FAK-mediated integrin signaling. Matrix Biol.

[CR49] Mosmann T (1983). Rapid colorimetric assay for cellular growth and survival: application to proliferation and cytotoxicity assays. J Immunol Methods.

[CR50] Niessner H, Kosnopfel C, Sinnberg T (2017). Combined activity of temozolomide and the mTOR inhibitor temsirolimus in metastatic melanoma involves DKK1. Exp Dermatol.

[CR51] Nishimichi N, Tsujino K, Kanno K (2021). Induced hepatic stellate cell integrin, α8β1, enhances cellular contractility and TGFβ activity in liver fibrosis. J Pathol.

[CR52] Novotný J, Strnadová K, Dvořánková B (2020). Single-cell RNA sequencing unravels heterogeneity of the stromal niche in cutaneous melanoma heterogeneous spheroids. Cancers.

[CR53] Peebles PT, Trisch T, Papageorge AG (1978). 727 isolation of four unusual pediatric solid tumor cell lines. Pediatr Res.

[CR54] Pfeffer S, Grossmann K, Cassidy P (2015). Detection of exosomal miRNAs in the plasma of melanoma patients. J Clin Med.

[CR55] Plzák J, Bouček J, Bandúrová V (2019). The head and neck squamous cell carcinoma microenvironment as a potential target for cancer therapy. Cancers.

[CR56] Preisner F, Leimer U, Sandmann S (2018). Impact of human adipose tissue-derived stem cells on malignant melanoma cells in an in vitro co-culture model. Stem Cell Rev.

[CR57] Quax PHA, van Muijen GNP, Weening-Verhoeff EJD (1991). Metastatic behavior of human melanoma cell lines in nude mice correlates with urokinase-type plasminogen activator, its type-1 inhibitor, and urokinase-mediated matrix degradation. J Cell Biol.

[CR58] R Core Team (2020) The R Project for Statistical Computing, Vienna, Austria. In: https://www.R-project.org/. Accessed 01 June 2021

[CR59] Rodemann HP, Rennekampff H-O (2011). Functional diversity of fibroblasts. Tumor-associated fibroblasts and their matrix.

[CR60] Schäfer M, Werner S (2008). Cancer as an overhealing wound: an old hypothesis revisited. Nat Rev Mol Cell Biol.

[CR61] Shao H, Kirkwood JM, Wells A (2015). Tenascin-C signaling in melanoma. Cell Adh Migr.

[CR62] Sjöqvist S, Ishikawa T, Shimura D (2019). Exosomes derived from clinical-grade oral mucosal epithelial cell sheets promote wound healing. J Extracell Vesicles.

[CR63] Smetana K, Lacina L, Szabo P (2016). Ageing as an important risk factor for cancer. Anticancer Res.

[CR64] Smetana K, Lacina L, Kodet O (2020). Targeted therapies for melanoma. Cancers.

[CR65] Stephens M (2017). False discovery rates: a new deal. Biostatistics.

[CR66] Théry C, Amigorena S, Raposo G, Clayton A (2006). Isolation and characterization of exosomes from cell culture supernatants and biological fluids. Curr Protoc Cell Biol.

[CR67] Todaro GJ, Green H (1963). Quantitative studies of the growth of mouse embryo cells in culture and their development into established linesJ. Cell Biol.

[CR68] Trotter MJ, Colwell R, Tron VA (2003). Thrombospondin-1 and cutaneous melanoma. J Cutan Med Surg.

[CR69] Tsering T, Laskaris A, Abdouh M (2020). Uveal melanoma-derived extracellular vesicles display transforming potential and carry protein cargo involved in metastatic niche preparation. Cancers.

[CR70] Valcz G, Buzás EI, Sebestyén A (2020). Extracellular vesicle-based communication may contribute to the co-evolution of cancer stem cells and cancer-associated fibroblasts in anti-cancer therapy. Cancers.

[CR71] Vlodavsky I, Korner G, Ishai-Michaeli R (1990). Extracellular matrix-resident growth factors and enzymes: possible involvement in tumor metastasis and angiogenesis. Cancer Metastasis Rev.

[CR72] Wang P, Zeng Z, Lin C (2020). Thrombospondin-1 as a potential therapeutic target: multiple roles in cancers. Curr Pharm Des.

[CR73] Webber J, Steadman R, Mason MD (2010). Cancer exosomes trigger fibroblast to myofibroblast differentiation. Cancer Res.

[CR74] Xu R, Greening DW, Rai A (2015). Highly-purified exosomes and shed microvesicles isolated from the human colon cancer cell line LIM1863 by sequential centrifugal ultrafiltration are biochemically and functionally distinct. Methods.

[CR75] Xu S, Xu H, Wang W (2019). The role of collagen in cancer: from bench to bedside. J Transl Med.

[CR76] Yao Z, Li J, Wang X (2020). Microrna-21-3p engineered umbilical cord stem cell-derived exosomes inhibit tendon adhesion. J Inflamm Res.

[CR77] Yates AD, Achuthan P, Akanni W (2020). Ensembl 2020. Nucleic Acids Res.

[CR78] Young MD, Wakefield MJ, Smyth GK, Oshlack A (2010). Gene ontology analysis for RNA-seq: accounting for selection bias. Genome Biol.

[CR79] Yu WY, Hill ST, Chan ER (2021). Computational drug repositioning identifies statins as modifiers of prognostic genetic expression signatures and metastatic behavior in melanoma. J Invest Dermatol.

[CR80] Zebrowska A, Widlak P, Whiteside T, Pietrowska M (2020). Signaling of tumor-derived sev impacts melanoma progression. Int J Mol Sci.

[CR81] Zhou Q, Wu X, Wang X (2020). The reciprocal interaction between tumor cells and activated fibroblasts mediated by TNF-α/IL-33/ST2L signaling promotes gastric cancer metastasis. Oncogene.

