# Supplementary figures and images for: Exosomes produced by melanoma cells significantly influence the biological properties of normal and cancer-associated fibroblasts
Source: Histochem Cell Biol. 2021 Nov 27;157(2):153–72. doi: 10.1007/s00418-021-02052-2 (PMC8847298; doi:10.1007/s00418-021-02052-2)

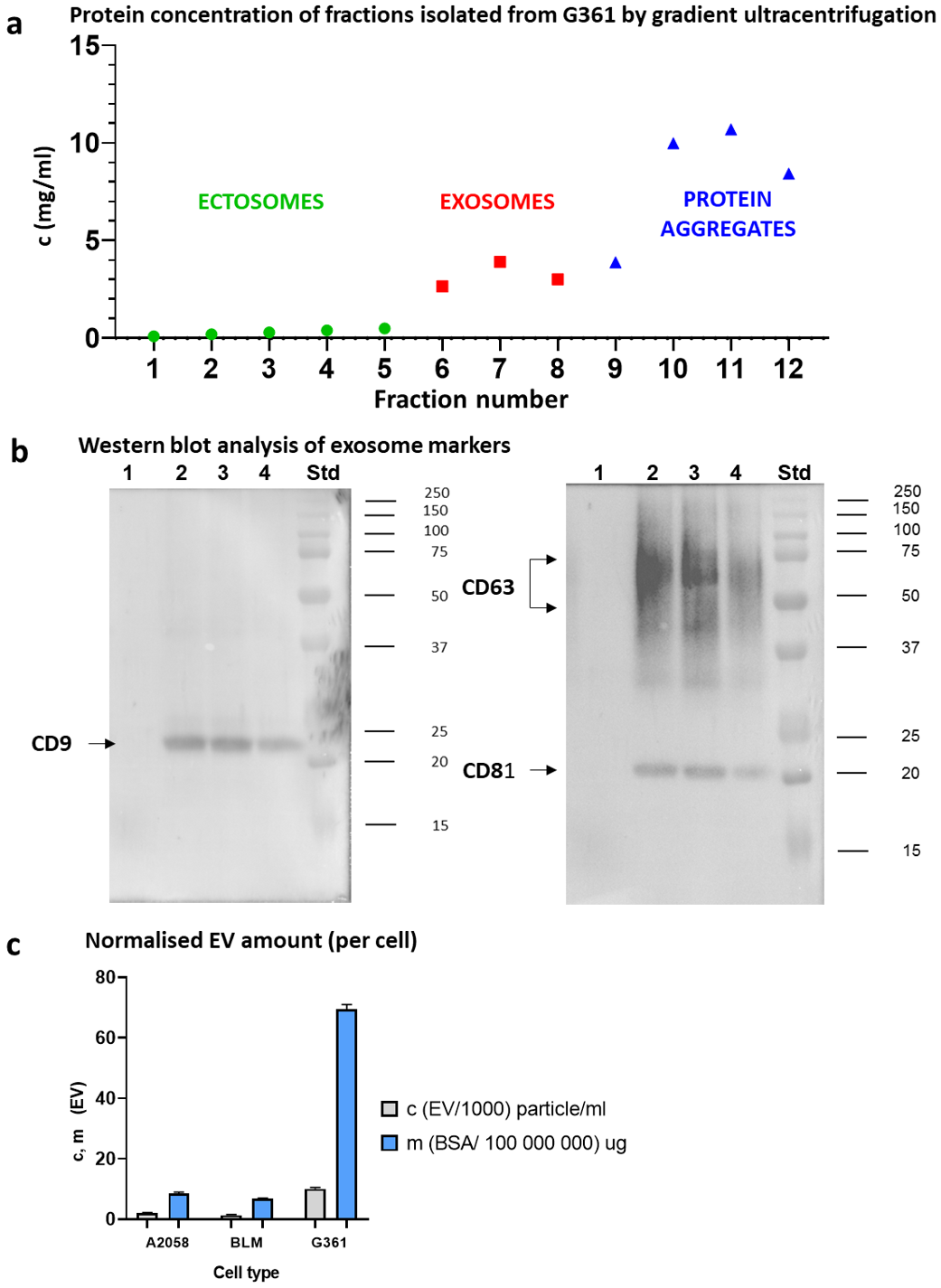

Supplement: Supplementary file 1 — (TIF 5805 kb) Protein fractions separated from G361 conditioned media. Western blot detection of exosome surface markers. Comparison of G361-isolated exosome number and protein concentration trend: (a) G361-derived extracellular vesicles were separated in fractions 1–12 by gradient ultracentrifugation. Exosomes were present in fractions 6–8. These fractions were further analysed by western blotting. (b) Exosome markers CD9, CD63 and CD81 were studied in: lane 1, 5% Thermo Scientific exosome-depleted FBS-enriched DMEM culture media (no band present), lanes 2–4 represent 5% Thermo Scientific exosome-depleted FBS-enriched DMEM supplemented by G361 self-prepared exosomes from fractions 6, 7 and 8, respectively. Bands with expected MW of CD9, CD63 and CD81 were detected. (c) Comparison of quantification of exosomes isolated by cushioned-density gradient ultracentrifugation. The number of cells (for normalisation) was counted after media harvesting using a haemocytometer. Concentration of exosomes (particles/mL) isolated from three melanoma cell lines were analysed by NTA using the NanoSight NS300 instrument and normalised (blue columns). Protein content in isolated exosomes was determined by BCA assay for WB protein loading (grey columns, represented in μg per 100,000,000 cells). Every column represents data from three independent experiments; error bars represent observed standard deviations. [file 418_2021_2052_MOESM1_ESM.tif]

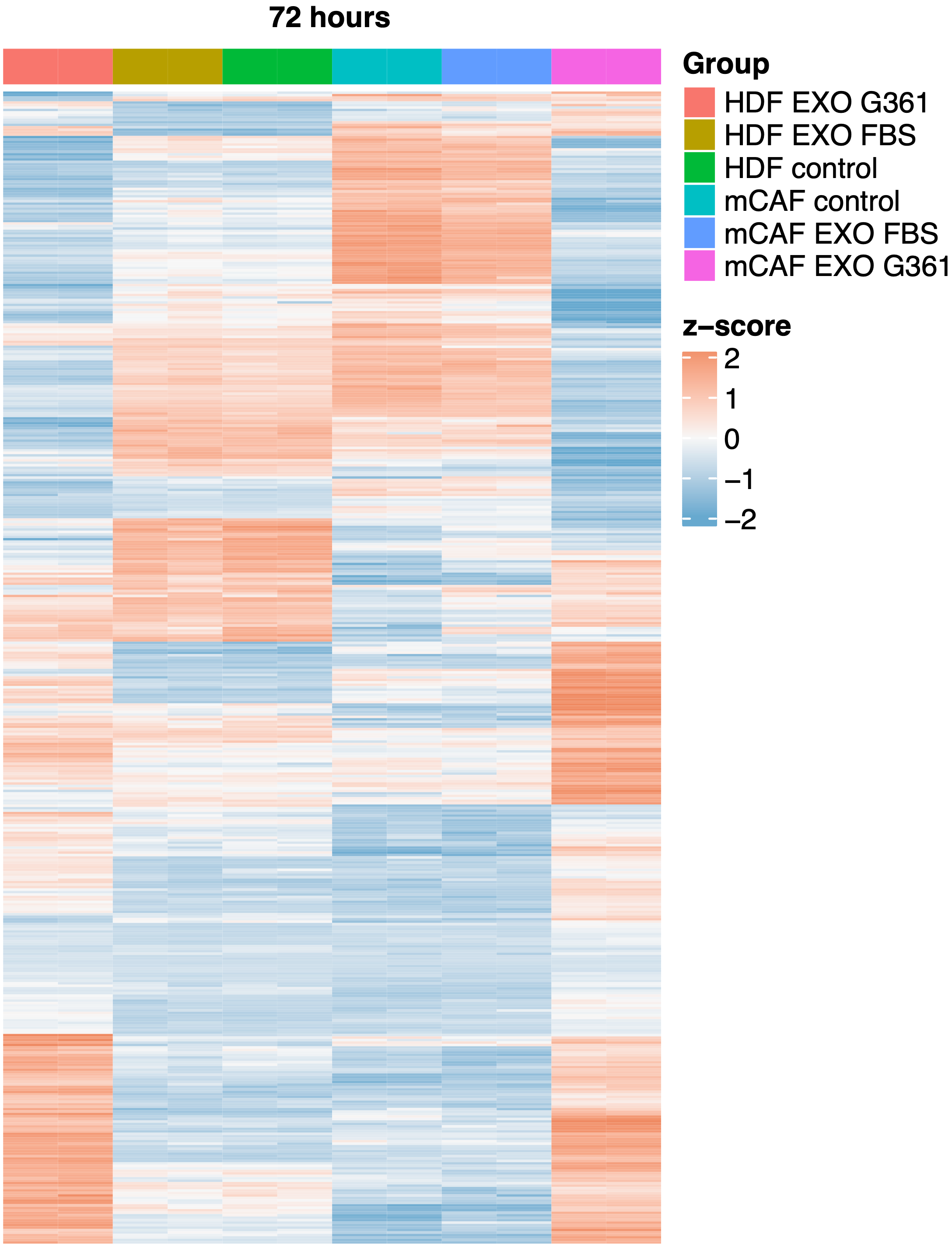

Supplement: Supplementary file 2 — (TIF 24273 kb) G-361-derived and to a lesser extent FBS-derived exosomes influenced the fibroblast transcriptome. The heatmap presents the transcriptomic profile of HDFs and mCAFs 72 hours after exosome stimulation. [file 418_2021_2052_MOESM2_ESM.tif]

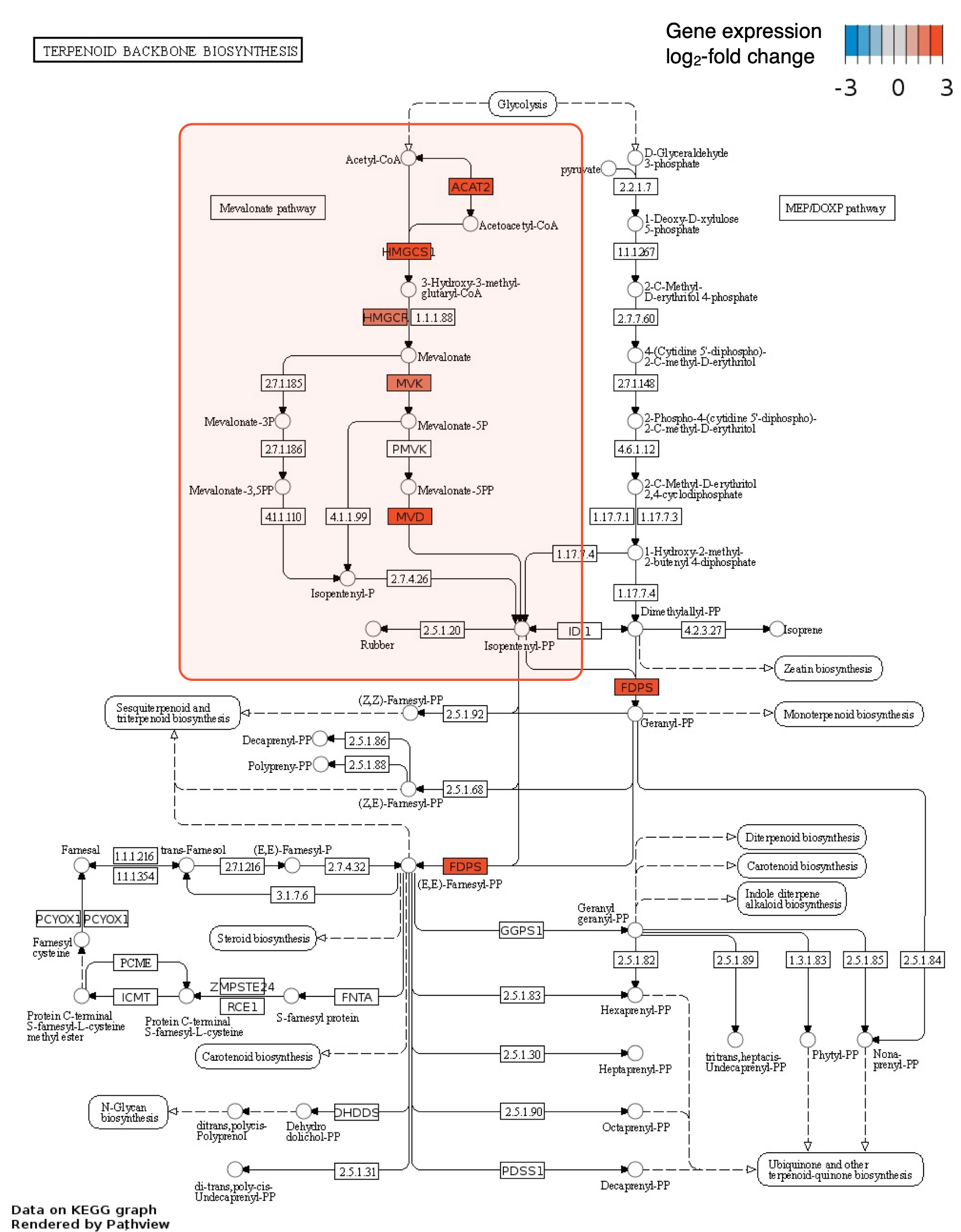

Supplement: Supplementary file 3 — (TIF 15273 kb) G-361-derived exosomes stimulate the mevalonate synthesis pathway in the studied fibroblasts. [file 418_2021_2052_MOESM3_ESM.tif]

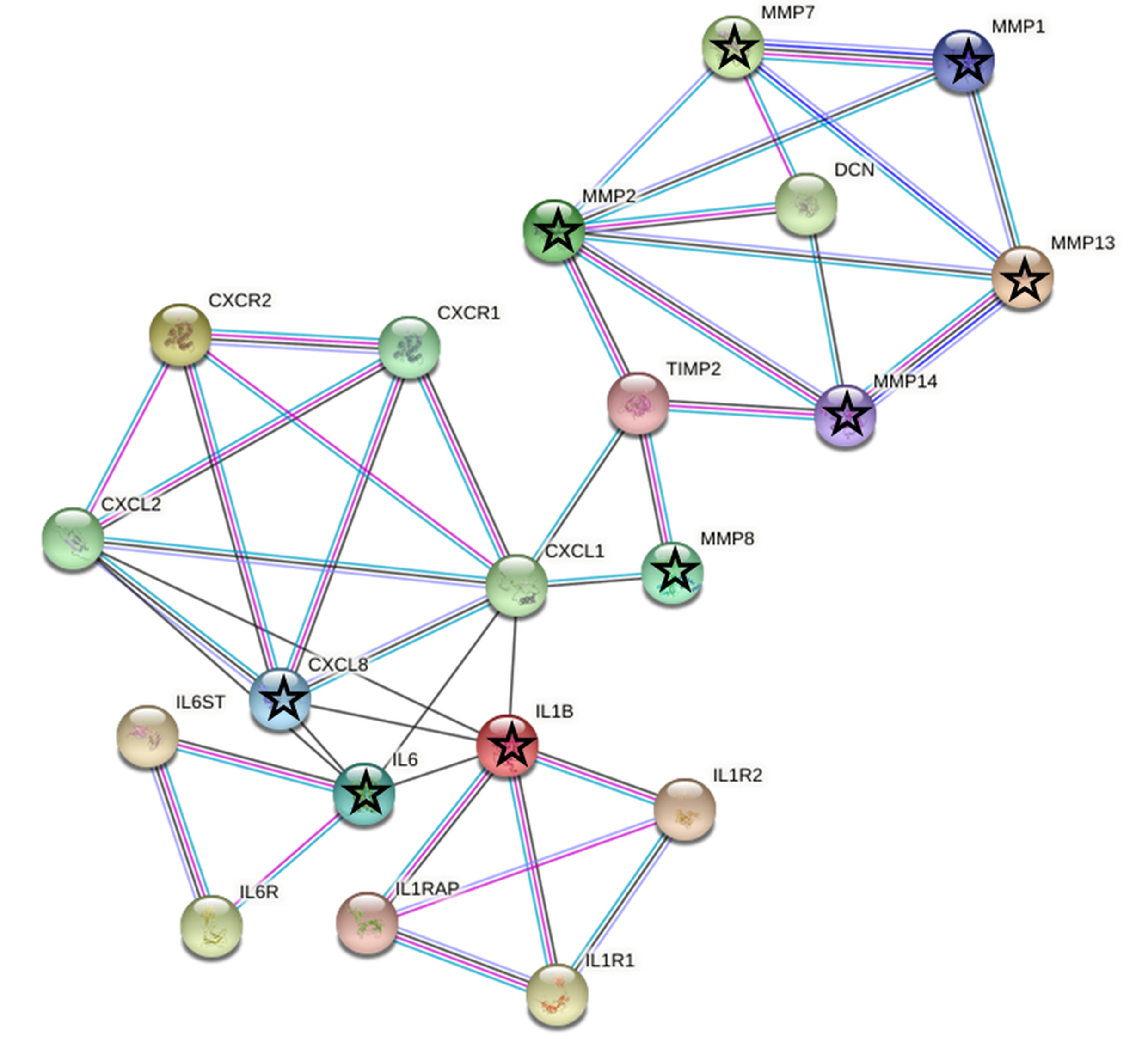

Supplement: Supplementary file 4 — (TIF 16372 kb) STRING database - Protein-Protein Interaction Networks Functional Enrichment Analysis. Functional link of MMPs with prominent proinflammatory molecules IL-1, IL-6 and CXCL-8 frequently expressed in mCAFs. STRING interaction network. https://string-db.org/cgi/network?taskId=bJAkTLRhTlxn&sessionId=bvZh9tqml4fP. Accessed 4 May 2021 [file 418_2021_2052_MOESM4_ESM.tif]

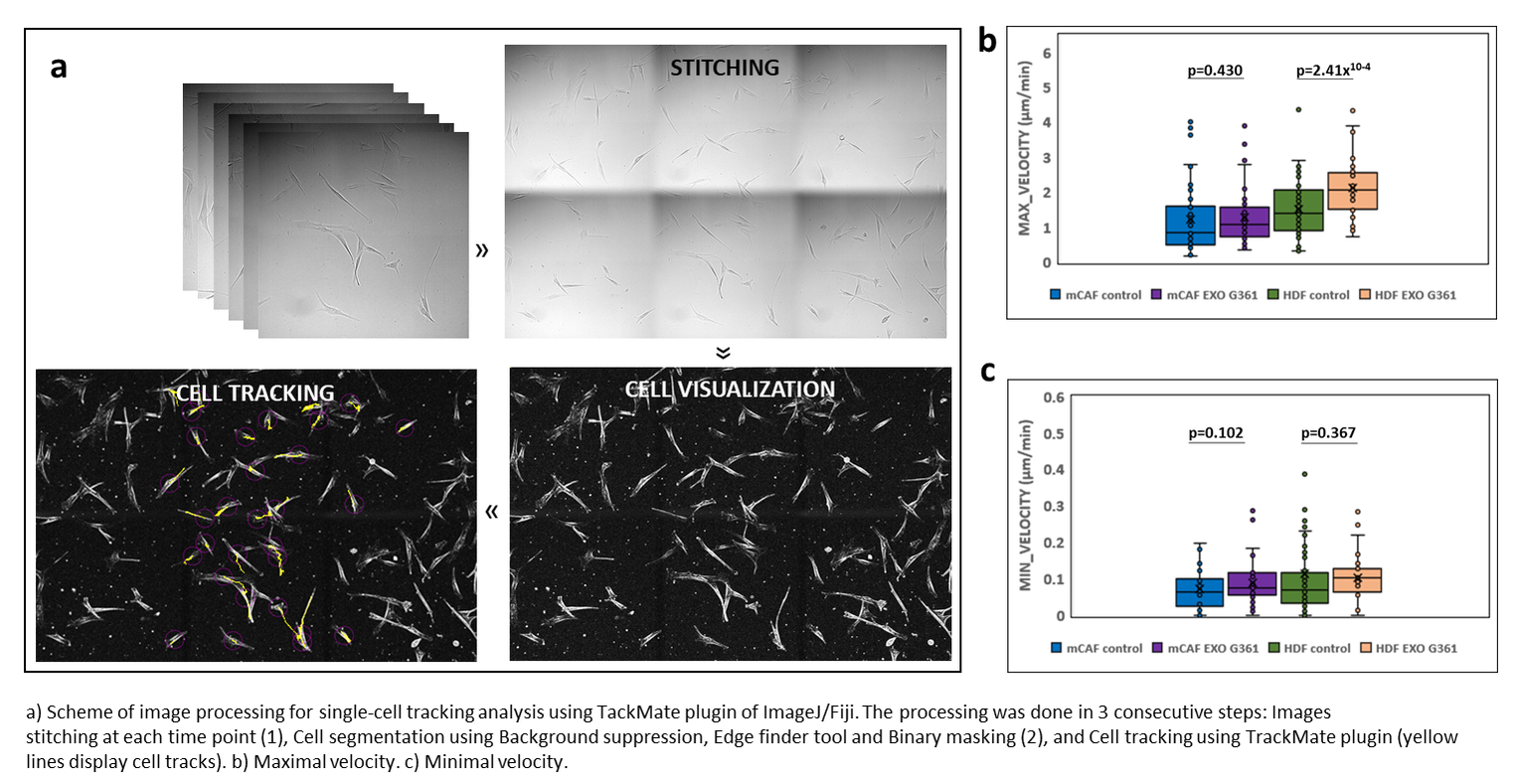

Supplement: Supplementary file 5 — (TIF 4703 kb) Cell tracking experiments workflow. a) Scheme of image processing for single-cell tracking analysis using TrackMate plug-in of ImageJ/Fiji. The processing was done in three consecutive steps: image stitching at each time point (1), cell segmentation using background suppression, Edge Finder tool and binary masking (2), and cell tracking using TrackMate plug-in (yellow lines display cell tracks). b) Maximal velocity. c) Minimal velocity [file 418_2021_2052_MOESM5_ESM.tif]
