# Supplementary material for: Exosomes produced by melanoma cells significantly influence the biological properties of normal and cancer-associated fibroblasts
Source: Histochem Cell Biol. 2021 Nov 27;157(2):153–72. doi: 10.1007/s00418-021-02052-2 (PMC8847298; doi:10.1007/s00418-021-02052-2)
Supplement: Supplementary file 6 — (DOCX 16 kb) [file 418_2021_2052_MOESM6_ESM.docx]

| **Target** | **Catalogue Nr.** | **Species/Type** | **Producer** | **Secondary antibody** |
| --- | --- | --- | --- | --- |
| - ***Westernblotting*** | | | | |
| CD9 | 10626D | Mouse monoclonal | Thermofisher Scientific | Goat anti-mouse IgG-HRP sc-2005  Santa Cruz Biotechnology |
| CD61 | 10628D | Mouse monoclonal | Thermofisher Scientific |  |
| CD83 | 10630D | Mouse monoclonal | Thermofisher Scientific |  |
| - ***Immunocytochemistry*** | | | | |
| IL-6 | ab9324 | Mouse monoclonal | ABCAM | Histofine® Simple Stain™ MAX PO (MULTI)  Nichirei Biosciences INC. |
| IL-6R | ab1280008 | Rabbit polyclonal | ABCAM |  |
| CXCL-8 | ab18672 | Mouse monoclonal | ABCAM |  |
| CXCR-1 | NAB330 | Mouse monoclonal | RD System |  |
| CXCR-2 | MAB331 | Mouse monoclonal | RD System |  |
| Isotype control Rabbit | 31235 | Rabbit Control | Thermofisher Scientific |  |
| Isotype control Mouse | 31903 | Mouse Control | Thermofisher Scientific |  |
| Antibody dilutent and blocking: Universal IHC Blocking/Diluent, LEICA PV6123 | | | | |
| Counterstaining: Gill's hematoxylin I – BAMED C0252 | | | | |
| Mounting: HYDROMOUNT™ Electron Microscopy Sciences - BIOGEN 17966 | | | | |
